# Faster-X Evolution of Gene Expression in *Drosophila*

**DOI:** 10.1371/journal.pgen.1003013

**Published:** 2012-10-11

**Authors:** Richard P. Meisel, John H. Malone, Andrew G. Clark

**Affiliations:** 1 Department of Molecular Biology and Genetics, Cornell University, Ithaca, New York, United States of America; 2 Department of Biological Science, Florida State University, Tallahassee, Florida, United States of America; University of California Berkeley, United States of America

## Abstract

DNA sequences on X chromosomes often have a faster rate of evolution when compared to similar loci on the autosomes, and well articulated models provide reasons why the X-linked mode of inheritance may be responsible for the faster evolution of X-linked genes. We analyzed microarray and RNA–seq data collected from females and males of six *Drosophila* species and found that the expression levels of X-linked genes also diverge faster than autosomal gene expression, similar to the “faster-X” effect often observed in DNA sequence evolution. Faster-X evolution of gene expression was recently described in mammals, but it was limited to the evolutionary lineages shortly following the creation of the therian X chromosome. In contrast, we detect a faster-X effect along both deep lineages and those on the tips of the *Drosophila* phylogeny. In *Drosophila* males, the dosage compensation complex (DCC) binds the X chromosome, creating a unique chromatin environment that promotes the hyper-expression of X-linked genes. We find that DCC binding, chromatin environment, and breadth of expression are all predictive of the rate of gene expression evolution. In addition, estimates of the intraspecific genetic polymorphism underlying gene expression variation suggest that X-linked expression levels are not under relaxed selective constraints. We therefore hypothesize that the faster-X evolution of gene expression is the result of the adaptive fixation of beneficial mutations at X-linked loci that change expression level in *cis*. This adaptive faster-X evolution of gene expression is limited to genes that are narrowly expressed in a single tissue, suggesting that relaxed pleiotropic constraints permit a faster response to selection. Finally, we present a conceptional framework to explain faster-X expression evolution, and we use this framework to examine differences in the faster-X effect between *Drosophila* and mammals.

## Author Summary

As species diverge over evolutionary time, they accumulate differences in the sequences of their genes and how those genes are expressed. We show that gene expression changes accumulate faster for genes on the X chromosome than for genes on the other chromosomes (autosomes) in *Drosophila* (the “faster-X” effect). The X chromosome is only found in a single copy in males, whereas the autosomes are found in two copies in both sexes. To compensate for the reduced dosage of X-linked genes in males, a molecular complex binds the *Drosophila* X chromosome to upregulate gene expression in males. We demonstrate that genes that escape this dosage compensation process have faster evolving expression levels. X-linked genes are inherited in a unique manner, and we hypothesize that this permits a faster rate of adaptive evolution, thereby driving the faster-X evolution of gene expression. We compare these observations with the recently described faster-X evolution of gene expression in mammals, and we explain how differences in dosage compensation, mutation rate, and population size could affect the extent of the faster-X effect.

## Introduction

Comparing the evolutionary rates of X-linked (or Z-linked) and autosomal genes can be informative of the nature of allelic dominance [1], the type of variation acted upon by natural selection [2], [3], the mutational process [4]–[8], and the effect of differences in population size on the efficacy of natural selection across taxa [9], [10]. Notably, DNA sequences on X (or Z) chromosomes often evolve faster than autosomal sequences (i.e., the “faster-X” effect). This may be a result of the adaptive fixation of recessive beneficial mutations in X-linked genes [1], [11]–[14], mutational biases associated with dosage compensation [15], or the smaller effective population size (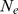) of sex chromosomes [9], [10]. The faster-X effect is especially pronounced in the protein coding sequences of genes with male-biased expression (i.e., genes expressed higher in males than females) or genes specifically expressed in male reproductive tissues in male heterogametic (XY) taxa [16]–[20]. These results support the theoretical prediction that the adaptive fixation of recessive X-linked male-beneficial mutations in hemizygous males can drive faster-X evolution [1].

Comparisons of expression divergence between X-linked and autosomal genes are not as prevalent as analyses of DNA sequences. Some experiments have suggested that the disproportionate effect of X-linked loci on interspecific hybrid fitness (the “large X” effect [21]) is the result of divergence in the regulation of gene expression. For example, gene expression from the X chromosome may be misregulated in the male germline of interspecific hybrids [22]–[24], and dosage compensation of the X chromosome could also be affected in hybrids [25]–[27]. With the advent of high throughput technologies to measure expression in multiple species we can now directly test whether the rate of expression evolution differs between X-linked and autosomal genes. The first such analysis did indeed find evidence for the faster-X evolution of gene expression shortly following the creation of the therian X chromosome [28].

Gene expression is determined by an interaction of *cis* regulatory elements and the proteins that bind to them (e.g., transcription factors, histones) to either promote or inhibit transcription. X chromosomes often have a unique chromatin environment because of the need to dosage compensate X-linked genes in males. In mammals, this is hypothesized to be accomplished by the upregulation of X-linked gene expression in both sexes, followed by random silencing of one X chromosome in females [29]–[31] (although this model is not universally accepted [32]). *Drosophila* compensate for reduced X chromosome dose in males by modifying the chromatin structure of the X in a male-specific manner. The dosage compensation complex (DCC; or male-specific lethal [MSL] complex), a ribonucleoprotein structure, binds the X chromosome in males, acetylating histone H4 at lysine 16 [33]–[35]. This is thought to promote the expression of X-linked genes via some combination of relaxing compacted chromatin [36], [37], enhancing recruitment of RNA polymerase II [38], and/or increasing transcriptional elongation [39]. The DCC only assembles in males because one of the essential proteins, MSL-2, is not produced in females [40]–[42].

Recently, chromatin immunoprecipitation (ChIP) experiments followed by microarrays (ChIP-chip) or sequencing (ChIP-seq) have revealed regions of the *Drosophila melanogaster* X chromosome that are enriched with DCC binding and bound by the DCC in the absence of essential DCC components [43], 44. These chromatin entry or high affinity sites (HASs) contain a DNA sequence motif that is thought to direct the DCC to the *Drosophila* X chromosome [43], [44]. After initially binding to the 100–300 HASs, the DCC is hypothesized to spread in *cis* to promote the upregulation of expression by inducing transcriptionally activating chromatin marks [45]–[49].

To examine how X-linkage, chromatin environment, and breadth of expression affect the evolution of gene expression, we calculated expression differences between *Drosophila* species using data collected from male and female whole flies and heads using microarrays and high throughput RNA sequencing (RNA-seq) [50]–[52]. We detect a robust signal of faster-X evolution of gene expression. This faster-X effect is most pronounced in genes that are located in transcriptionally repressive chromatin in cell culture and genes that are narrowly expressed in a limited number of tissues. In addition, we analyzed measurements of intraspecific variation in gene expression, and we found that the faster-X effect cannot be explained by relaxed selective constraints. Our results suggest that the faster-X evolution of gene expression is the result of the adaptive fixation of X-linked mutations that affect gene expression in *cis*.

## Results

### Faster-X evolution of gene expression

**Figure 1:**
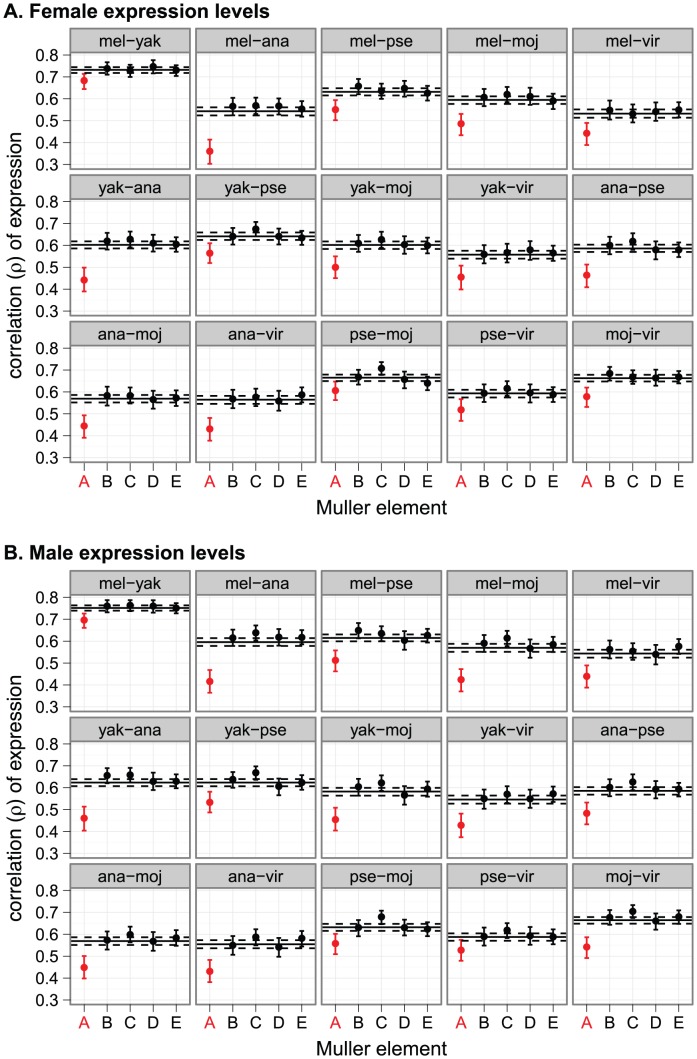
Faster-X evolution of gene expression. Pairwise correlations of gene expression are shown for genes on each chromosome arm, using expression measurements from (A) females or (B) males. In each graph, the solid horizontal line is the genome-wide correlation, and the dashed lines are the 95% confidence interval (CI). Each point represents the correlation for a chromosome arm, and the error bars are the 95% CI. Chromosome arms are represented with their Muller element nomenclature. Muller element A is is the X chromosome (red), and Muller element D is the *D. pseudoobscura* neo-X chromosome. Species names were abbreviated as follows: mel =  *D. melanogaster*, yak =  *D. yakuba*, ana = *D. ananassae*, pse = *D. pseudoobscura*, moj = *D. mojavensis*, vir = *D. virilis*.

We analyzed expression measurements in six *Drosophila* species (*D. melanogaster*, *Drosophila yakuba*, *Drosophila ananassae*, *Drosophila pseudoobscura*, *Drosophila mojavensis*, and *Drosophila virilis*) collected from whole females and males separately using microarrays. Following the method of Brawand et al. [28], we calculated the similarity in expression between pairs of species, within each sex, using Spearman's rank correlation coefficient (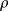) sampling only genes present as 1∶1∶1∶1∶1∶1 orthologs across all six species [53]. To determine if the expression levels of X-linked genes diverge faster than autosomal genes, we compared 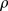 across the five major chromosome arms (also known as Muller elements [54]). In every pairwise comparison, the correlation of expression levels of X-linked genes (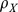), in both females and males, is significantly lower than that of autosomal genes (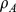) (Figure 1). We confirmed this pattern using a different pipeline to handle the microarray data (Figure S1) and with RNA-seq data from three species (*D. melanogaster*, *D. pseudoobscura*, and *D. mojavensis*; Figure S2). These results suggest that X-linked gene expression levels diverge faster than the expression levels of autosomal genes.

While there is congruence between the microarray and RNA-seq data with regards to the faster-X evolution of expression, we observe two notable differences between these data sets. First, expression levels estimated using RNA-seq are more highly correlated than those estimated from microarray data (Figure 1, Figure S2), possibly because of the increased dynamic range of RNA-seq [55]. Second, the magnitude of the difference between 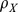 and 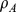 is greater in the microarray data than the RNA-seq data (Figure 1, Figure S2). We reanalyzed the microarray data using only the genes present in the RNA-seq dataset, and these correlations resemble the microarray analysis more than the RNA-seq (Figure S3). Therefore, the difference in magnitude of 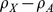 in the microarray and RNA-seq analyses is not attributable to differences in the gene sets analyzed. Regardless of the cause of these differences, both methodologies provide evidence in support of the faster-X evolution of gene expression (Figure 1, Figure S2).

### Faster-X expression evolution is not limited to genes with male-biased expression or new genes

**Figure 2:**
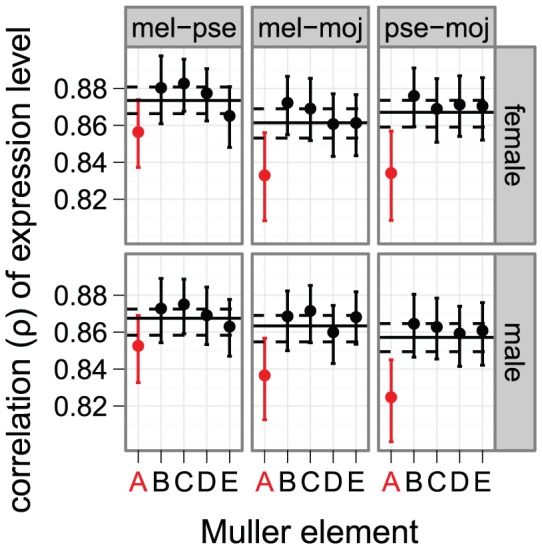
Faster-X evolution of gene expression measured in head. Gene expression measurements from head using microarrays were used to calculate the correlation of expression levels between *D. melanogaster* (mel), *D. pseudoobscura* (pse), and *D. mojavensis* (moj). Expression was measured in females (top) and males (bottom). Graphs are the same as in Figure 1.

The faster-X evolution of *Drosophila* protein coding sequences is especially pronounced in genes with male-biased expression that are expressed in male reproductive tissues [18]–[20], possibly because the hemizygosity of the X chromosome favors the adaptive fixation of recessive male-beneficial mutations in X-linked genes [1]. Additionally, genes with male-biased expression tend to have more divergent expression between species than genes with female-biased or non-sex-biased expression [56], [57]. The faster-X evolution of gene expression, however, is not limited to genes expressed in male reproductive tissues: we detect the faster-X effect when gene expression is measured in females (Figure 1) or heads (Figure 2, Figure S4), although the pattern is not as striking as when whole fly data are used.

**Figure 3:**
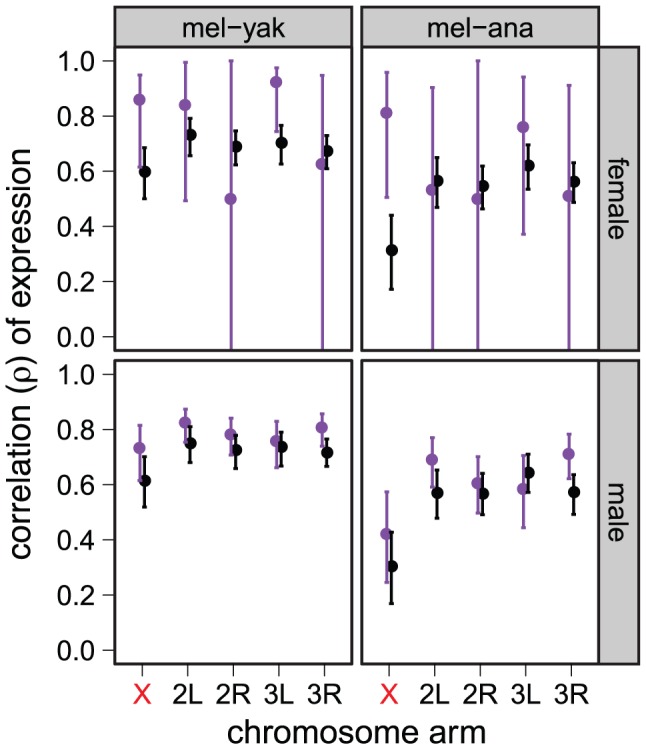
Faster-X expression evolution for genes expressed in male, but not female, reproductive tissues. Correlations of expression between *D. melanogaster* (mel) and either *D. yakuba* (yak) or *D. ananassae* (ana) are plotted for each chromosome arm using expression measurements from whole females (top) or males (bottom). Genes were included if they are narrowly expressed in non-reproductive tissues (black), female reproductive tissues (purple, top), or male reproductive tissues (purple, bottom). Graphs are the same as in Figure 1.

To further examine the effect of expression in male-reproductive tissues on the faster-X effect, we excluded genes with male-biased expression in *D. melanogaster* (765 genes at a false discovery rate [FDR] of 0.05), male-biased expression in all of the species (2027 genes at a FDR of 0.20), or biased expression in male reproductive tissues in *D. melanogaster* (439 genes based on expression data from FlyAtlas [58]). In all cases, we detect the faster-X evolution of gene expression even when genes with male-biased expression are removed (Figures S5, S6, S7). In addition, genes that are narrowly expressed in non-reproductive tissues exhibit a faster-X effect comparable to genes with biased expression in male reproductive tissues (Figure 3). On the other hand, we fail to detect the faster-X effect when we consider only genes with biased expression in female-limited reproductive tissues (Figure 3). We therefore conclude that the faster-X evolution of gene expression requires expression in males but not necessarily male-biased expression.

Genes that arose recently by duplication tend to have male-biased expression [59], [60]. Many new *Drosophila* genes are located on the X chromosome and show evidence of a faster-X effect in their protein coding sequences [61]. We find that new genes (those that arose after the split between the *D. melanogaster* and *D. virilis* lineages) do not exhibit evidence of faster-X expression evolution, while old genes shared by the entire genus do (Figure S8). Therefore, the faster-X evolution of male reproductive genes, genes with male-biased expression, or new genes are not entirely responsible for the faster-X evolution of gene expression in *Drosophila*.

### Faster-X expression evolution along both internal and tip lineages of the *Drosophila* phylogeny

Previous work in mammals found evidence for faster-X evolution of gene expression that was limited to evolutionary lineages closely following the creation of the therian X chromosome [28]. To test for a similar lineage-specific faster-X effect in *Drosophila*, we used our calculations of 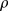 from whole fly expression measurements between species to estimate branch lengths along the *Drosophila* phylogeny. This approach assumes that there is a phylogenetic signal in these pairwise correlations. Some pairwise correlations are lower for more closely related species than more distantly related ones (Figure 1), suggesting that the correlations may not have an underlying phylogenetic signal. To further test for phylogenetic signal, we estimated the divergence in expression between species as 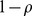 [28]. We were indeed able to reconstruct the evolutionary relationships of the six species using this distance matrix (Figure S9), demonstrating that the expression correlations contain a phylogenetic signal.

**Figure 4:**
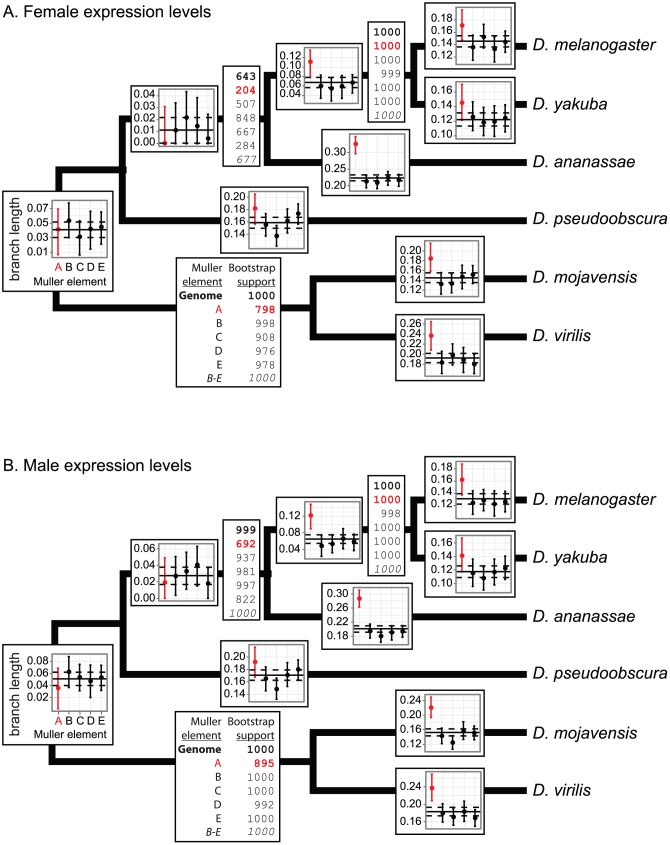
Branch length estimates and bootstrap support from expression level divergence. Overlaid on each branch of the phylogeny are the branch lengths estimated from the pairwise correlations in expression (measured in whole flies with microarrays) using the Fitch and Margoliash [115] method. In each graph, the solid horizontal line is the genome-wide branch length, and the dashed lines are the 95% CI. Each point represents the branch length estimate for a chromosome arm (X is in red), and the error bars are the 95% CI. Bootstrap supports for the nodes are listed in the boxes adjacent to the nodes. The first number, in bold, is the bootstrap support using all orthologs, and the subsequent five values are for genes on each chromosome arm (X is in red). The last value, in italics, is the bootstrap support using genes on the four autosomes (Muller elements B–E). Chromosome arms are represented with their Muller element nomenclature, as described in Figure 1. The Fitch and Margoliash algorithm treats the phylogeny as unrooted, so there is a single branch length and bootstrap value for the lineage connecting the Sophophora (*D. melanogaster*, *D. yakuba*, *D. ananassae*, and *D. pseudoobscura*) and Drosophila (*D. mojavensis* and *D. virilis*) subgenera. Branch lengths and bootstrap support were estimated using expression measurements from (panel A) females and (panel B) males.

We next tested whether the faster-X evolution of gene expression is limited to particular branches in the *Drosophila* phylogeny using matrices of 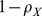 and 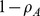 to estimate branch lengths along the known topology (Figure 4). These branch lengths represent the contribution that each lineage makes toward 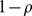, and larger values indicate a lower correlation in expression. Phylogenies constructed using X-linked gene expression from both males and females have longer internal and terminal branch lengths (Figure 4), unlike in mammals where a faster-X effect is only detected on internal branches [28].

Interestingly, branch length estimates closest to the root do not show evidence for a faster-X effect in *Drosophila* (Figure 4). This is not necessarily evidence against the faster-X evolution of gene expression along these internal branches. We instead hypothesize that it is the result of low power to resolve deep branching orders using these correlation matrices, which leads to poor estimates of branch lengths around deep nodes. Supporting this hypothesis, when we use the correlation matrices to estimate the tree topology, some of the deep nodes have the lowest bootstrap support for the correct topology (Figure 4, Figure S9). In addition, the bootstrap support for these nodes is lower for X-linked gene expression levels than autosomal expression (Figure 4). Furthermore, when we exclude genes on the X chromosome, we observe an increase in bootstrap support for the correct branching order between *D. pseudoobscura* and the *melanogaster* group (Figure 4, bottom number in bootstrap boxes, in italics). We therefore hypothesize that the faster-X evolution of gene expression complicates the inference of the correct branching order more for X-linked genes than autosomal genes at these deep nodes, leading to a flawed measurement of the branch lengths. In summary, depending on the branch in question, either longer branch lengths or lower bootstrap values support the hypothesis that X-linked gene expression levels diverge faster than autosomal expression levels across most of the phylogeny.

*D. pseudoobscura* and *Drosophila willistoni* each have an independently derived neo-X chromosome arm (Muller element D) that is autosomal in all other species [62]. If the faster evolution of gene expression closely follows the creation of an X chromosome, we would expect to detect a faster-X effect in genes on these neo-X chromosome arms. Using available RNA-seq data collected from *D. pseudoobscura*, *D. willistoni*, *D. melanogaster*, and *D. mojavensis* heads [52], we find some evidence for the faster evolution of gene expression on the neo-X chromosome arms (Figure S4). However, we fail to detect evidence for faster-X expression evolution in genes on the *D. pseudoobscura* neo-X chromosome when expression is measured in whole fly (Figure 1, Figure 4). The latter result may be because of low power to detect a faster-neo-X effect; the X-autosome fusion giving rise to the *D. pseudoobscura* neo-X occurred recently relative to the *pseudoobscura*-*melanogaster* common ancestor [60], [63].

### Faster-X expression evolution of genes unbound by the DCC and further from HASs

The *Drosophila* X chromosome has a unique chromatin environment because of the need to compensate dosage in hemizygous males [33], [34], [36], [64], and these histone modifications are correlated with gene expression levels [65]. We therefore considered whether DCC binding is associated with the faster-X effect. To do so, we calculated pairwise expression divergence for each 1∶1∶1 ortholog between *D. melanogaster*, *D. yakuba*, and *D. ananassae*. We selected these three closely related species because DCC binding and chromatin states have only been inferred for *D. melanogaster* [45], [46], and these inferences are less likely to be accurate in more distantly related species. In addition, these gene-wise estimates of expression divergence differ from the correlations in expression levels across entire chromosomes (see Methods). We chose this approach because, as we introduce more parameters into our analysis, gene-wise expression divergence is easier to interpret than correlations of chromosome-wide expression between species.

**Figure 5:**
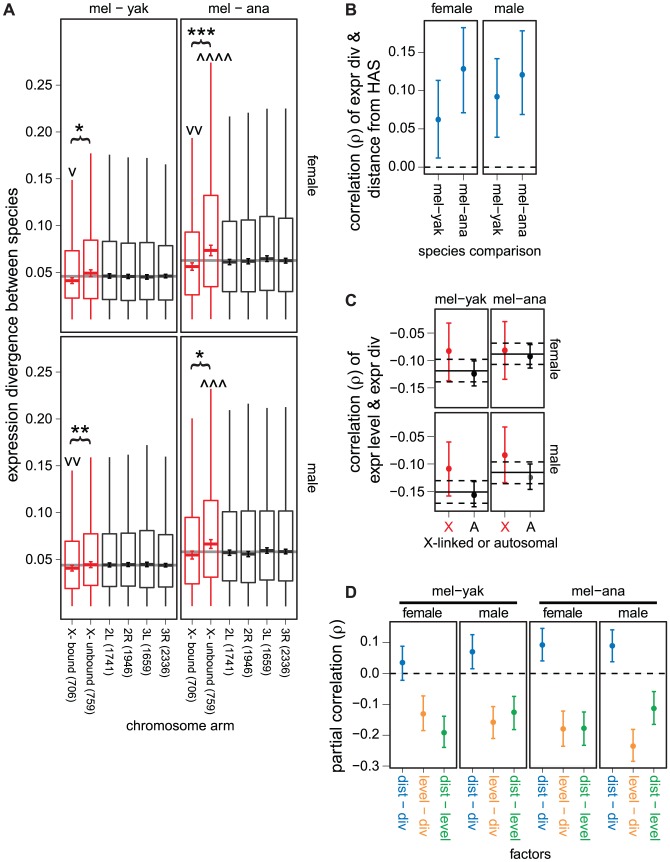
Faster expression evolution of X-linked genes not directly regulated by the DCC. (A) Boxplots show the pairwise divergence in expression between 1∶1∶1 orthologs in the *D. melanogaster* (mel), *D. yakuba* (yak), and *D. ananassae* (ana) genomes measured in whole females (top) and males (bottom) on each chromosome arm. X-linked genes are divided into those that are bound and unbound by the DCC. The counts of genes on each chromosome and DCC bound and unbound genes are given along the x-axis. Boxes extend from the first to the third quartile (interquartile range; IQR), the horizontal line in the middle of the box indicates the median value, and the whiskers represent 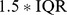 (outliers are not plotted). Error bars within each box show the location of 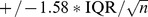 (where 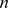 is the sample size), which approximates a 95% CI of the median. The genome-wide average is represented by the horizontal gray line. X-linked genes whose median pairwise divergence is greater () or less (v) than autosomal genes are marked (there is not a significant difference in expression divergence between autosomes). Asterisks indicate significant differences in the medians between X-linked genes bound and unbound by the DCC. Mann-Whitney U tests were used to assess significant differences (one symbol = 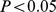, two symbols = 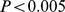, three symbols = 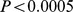, four symbols = 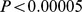). (B) Plots show the correlation between distance from the nearest HAS and the pairwise expression divergence between *D. melanogaster* (mel) and *D. yakuba* (yak) or *D. ananassae* (ana), along with the 95% CI. Expression levels were measured in females (top) and males (bottom). The dashed horizontal line shows the null expectation (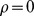). (C) Plots show the correlation between expression level in *D. melanogaster* and expression divergence between mel and yak or ana for autosomal (A) and X-linked (X, red) genes, along with the 95% CI. Expression levels were measured in females (top) and males (bottom). The solid horizontal line is the genome-wide correlation, and the dashed lines are the 95% CI of the genome-wide value. (D) Plots show the partial correlations between distance from the nearest HAS (dist), pair-wise divergence in expression between 1∶1∶1 orthologs (div), and expression level in *D. melanogaster* (level). Error bars represent the 95% CI of the partial correlations, estimated by bootstrapping. The dashed horizontal line shows the null expectation (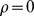).

Using the gene-wise estimates of expression divergence between *D. melanogaster* and either *D. yakuba* or *D. ananassae*, we found that X-linked genes that are unbound by the DCC have greater expression divergence than DCC bound X-linked genes (Figure 5A). Additionally, in the comparison between *D. melanogaster* and *D. ananassae*, unbound X-linked genes have greater expression divergence than autosomal genes (Figure 5A). Furthermore, the expression levels of DCC bound X-linked genes are more evolutionarily conserved than autosomal genes (Figure 5A). DCC bound genes tend to be in close proximity to HASs [48], and HASs have the highest concentration of DCC binding [43], suggesting that proximity to an HAS may also predict expression divergence. Distance from the nearest HAS is indeed positively correlated with expression divergence (Figure 5B). We observe these patterns when expression is measured in either females or males (Figure 5A–5B).

Highly expressed genes tend to have more conserved protein coding sequences [19], [66], and there may be a positive correlation between the rate of protein coding sequence evolution and divergence in gene expression [67]–[69]. Genes bound by the DCC have higher expression levels than unbound genes [48], suggesting that the relationship between DCC binding and expression divergence (Figure 5A–5B) may be a byproduct of highly expressed genes with less expression divergence. We found a negative correlation between expression level and expression divergence for both X-linked and autosomal genes (Figure 5C), demonstrating that highly expressed genes have more conserved expression levels.

To test whether the relationship between DCC binding and expression divergence is merely an artifact of the correlation between expression level and expression divergence, we calculated partial correlations between expression divergence, distance from the nearest HAS, and expression level. If DCC binding and expression divergence are directly related, genes further from an HAS should have elevated expression divergence even when expression level is taken into account. Distance from the nearest HAS is positively correlated with expression divergence in most of our partial correlations (Figure 5D), demonstrating that genes that are not directly regulated by the DCC have faster evolving expression levels. In addition, expression level and expression divergence are negatively correlated (Figure 5D), supporting the hypothesis that highly expressed X-linked genes have more conserved expression levels independent of DCC binding. Lastly, distance from an HAS is negatively correlated with expression level (Figure 5D), providing additional evidence that highly expressed genes are more directly regulated by the DCC [48].

### Faster expression evolution of X-linked genes located in transcriptionally repressive chromatin

The DCC is both attracted to and promotes chromatin modifications associated with transcriptional activity [35], [49], suggesting that genes unbound by the DCC are in transcriptionally repressive chromatin. The faster expression evolution of X-linked genes that are unbound by the DCC could therefore be a general property of genes associated with repressive chromatin. To test this hypothesis, we obtained mapped chromatin states in the *D. melanogaster* genome from two different cell lines [65], and we used these data to assign genes to one of two chromatin states: transcriptionally active or repressive. We found that X-linked genes that are bound by the DCC are indeed almost always (97.8–100%) associated with active chromatin, while unbound genes tend to be in repressive chromatin (Table S1). In addition, genes in transcriptionally active chromatin have higher expression levels than genes in repressive chromatin (Figure S10).

**Figure 6:**
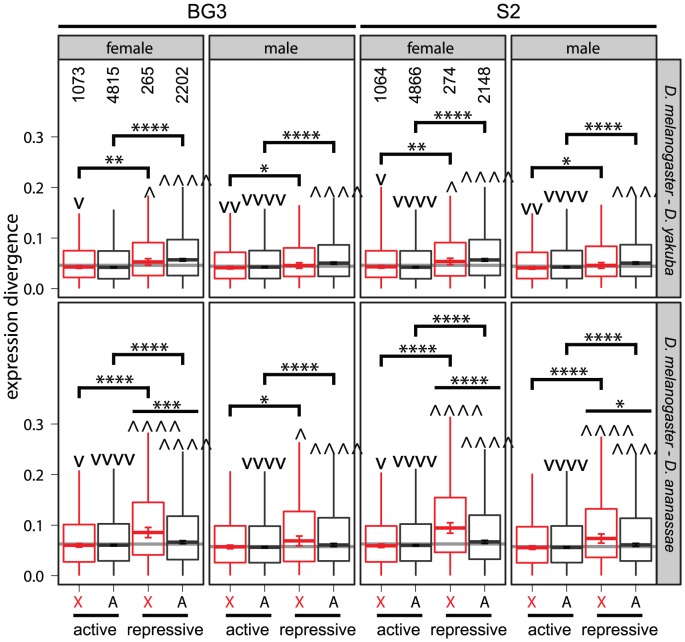
Faster expression evolution of genes associated with transcriptionally repressive chromatin. Boxplots show the pairwise divergence in expression between 1∶1∶1 orthologs in the *D. melanogaster* and *D. yakuba* or *D. ananassae* genomes measured in whole females and males (see Figure 5). X-linked (X, red) and autosomal (A) genes were assigned to transcriptionally active and repressive chromatin based on the results of ChIP-chip experiments in one of two cell lines (BG3 and S2) [65]. Counts of genes in each chromatin state are given for data collected from each of the cell lines in the top left quadrants. Groups of genes whose pairwise divergence is greater () or less (v) than the rest of the genome are marked. Comparisons were also made between genes in active and repressive chromatin on the X chromosome or autosomes, and comparisons were made between autosomal and X-linked genes in active or repressive chromatin. Mann-Whitney U tests were used to assess significant differences (one symbol = 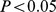, two symbols = 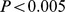, three symbols = 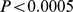, four symbols = 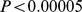).

Both autosomal and X-linked genes associated with transcriptionally repressive chromatin have more divergent expression levels than genes associated with active chromatin regardless of which cell type is used to infer chromatin states (Figure 6). However, X-linked genes that are located in repressive chromatin have more divergent expression between *D. melanogaster* and *D. ananassae* than autosomal genes in repressive chromatin (Figure 6). Furthermore, X-linked genes associated with active chromatin tend to have less expression divergence than other genes in the genome (Figure 6). We observe similar patterns when we use DCC binding as a proxy for transcriptionally active chromatin in X-linked genes (Figure S11). These results provide further support for the hypothesis that the faster-X evolution of gene expression is driven by genes that are not directly regulated by the DCC.

### Expression breadth, chromatin environment, and the faster-X effect

**Figure 7:**
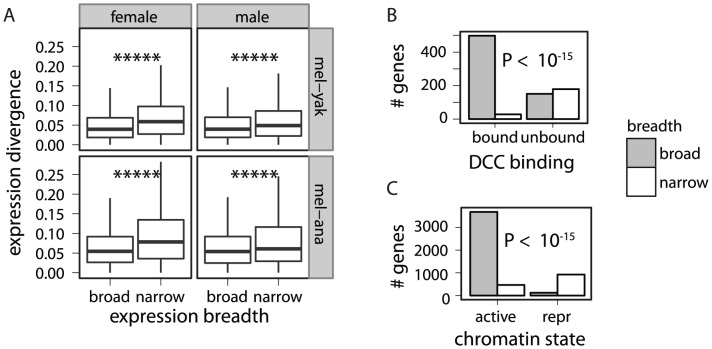
Association between expression breadth, DCC binding, chromatin state, and expression divergence. Genes were classified based on their expression breadth, whether they are bound by the DCC, and whether they are in transcriptionally active or repressive (repr) chromatin (based on data from S2 cells). (A) Boxplots show the pairwise divergence in expression between 1∶1∶1 orthologs in the *D. melanogaster* (mel) and *D. yakuba* (yak) or *D. ananassae* (ana) genomes for broadly and narrowly expressed genes (see Figure 5). Mann-Whitney U tests were used to assess significant differences in expression divergence between broadly and narrowly expressed genes (***** 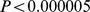). (B) X-linked broadly expressed (gray) and narrowly expressed (white) genes were divided into those that are bound and unbound by the DCC. (C) Broadly expressed (gray) and narrowly expressed (white) genes were divided into those that are in transcriptionally active and repressive chromatin. (B–C) Fisher's exact test was used to determine if there is a non-random distribution of genes in the four classes.

Genes expressed narrowly (i.e., in a limited number of tissues) tend to have rapidly evolving protein coding sequences [19], [66], which raises the possibility that expression breadth may also affect expression divergence. We find that narrowly expressed genes do tend to have elevated expression divergence (Figure 7A, Figure S12). In addition, DCC bound genes tend to be broadly expressed, and genes further from an HAS are more narrowly expressed (Figure 7B, Figure S13). Furthermore, genes that are in transcriptionally active chromatin tend to be broadly expressed, while genes in repressive chromatin tend to be narrowly expressed (Figure 7C, Figure S14). This raises the possibility that the association between chromatin environment and the faster-X effect (Figure 6) could be an artifact of the correlation between expression breadth and expression divergence.

**Figure 8:**
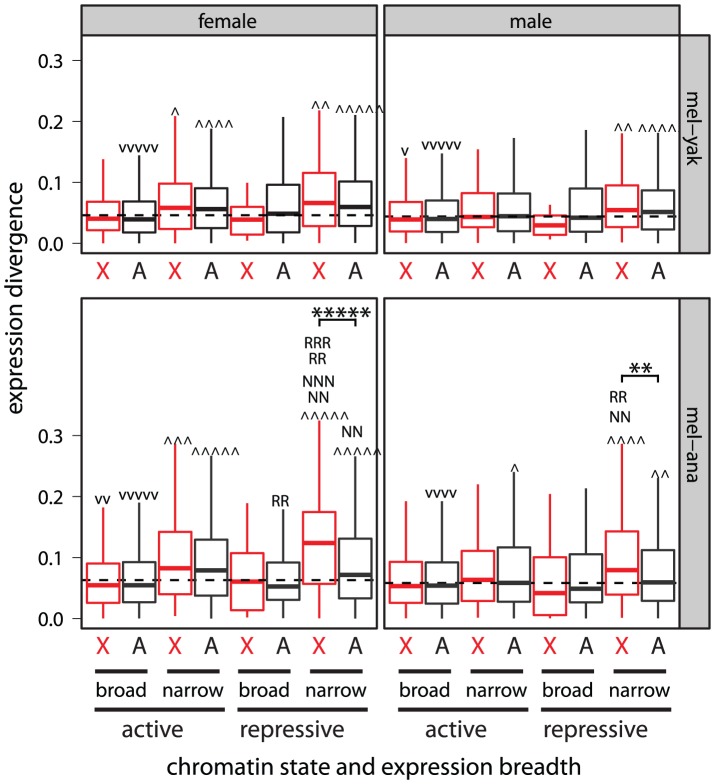
The faster-X effect is limited to narrowly expressed genes in transcriptionally repressive chromatin. Boxplots show the pairwise divergence in expression between 1∶1∶1 orthologs in the *D. melanogaster* (mel) and *D. yakuba* (yak) or *D. ananassae* (ana) genomes measured in whole females and males (see Figure 5, Figure 6). X-linked (X, red) and Autosomal (A) genes were assigned to transcriptionally active and repressive chromatin based on the results of experiments in S2 cells (for the results from BG3 cells see Figure S15). Groups of genes whose pairwise divergence is greater 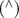 or less (v) than the rest of the genome are marked. Subsets of narrowly expressed genes whose pairwise divergence is significantly different than all other narrowly expressed genes are marked (N). The same was done for subsets of genes in repressive chromatin (R). Significant differences between X-linked and autosomal genes in the same chromatin state and with the same expression breadth are marked with asterisks. Mann-Whitney U tests were used to assess significant differences (one symbol = 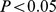, two symbols = 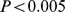, three symbols = 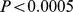, four symbols = 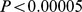, five symbols = 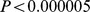).

If the faster-X evolution of gene expression is affected by expression breadth and not chromatin environment, we expect to only detect the faster-X effect in narrowly expressed genes. Consistent with this prediction, we detect the strongest evidence for faster-X evolution in narrowly expressed genes (Figure 8, Figure S15). The faster-X effect is, however, limited to narrowly expressed genes in transcriptionally repressive chromatin (Figure 8, Figure S15), suggesting that narrow expression and transcriptionally repressive chromatin environment both promote faster-X expression evolution. Narrowly expressed genes in transcriptionally repressive chromatin are more likely to have low expression levels [19], [66] (Figure S10), which could increase the error in expression level measurements. However, measurement error is unlikely to explain the association of expression breadth and chromatin environment with the faster-X effect for two reasons. First, experimental and biological variance should not produce the consistent signal of faster-X evolution. Second, we still detect the faster-X effect when genes with low expression levels are excluded (Figure S16). x

The faster-X effect could be a result of differences in gene content between the X chromosome and the autosomes if, for example, X-linked genes were more narrowly expressed or more likely to be in transcriptionally repressive chromatin than autosomal genes. The *D. melanogaster* X chromosome, however, harbors a deficiency of narrowly expressed genes [52], [70], and there is a paucity of X-linked genes in repressive chromatin (Figure 6; 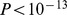 using Fisher's exact test). In addition, we fail to detect a significant difference in expression breadth between X-linked and autosomal genes in repressive chromatin (Figure S17). It is therefore unlikely that the unique gene content of the *Drosophila* X chromosome is responsible for the faster-X effect. Our power to detect the faster-X effect is also limited by the small sample size of narrowly expressed genes in repressive chromatin on the X chromosome, demonstrating that our results are conservative.

The faster-X evolution of protein-coding sequences is most pronounced for genes that are narrowly expressed in male reproductive tissues [19], [20]. We showed, however, that expression in male reproductive tissues is not solely responsible for the faster-X evolution of gene expression (Figure 2, Figure 3; Figures S4, S5, S6, S7). This does not exclude the possibility that X-linked genes expressed in male reproductive tissues have faster evolving expression levels than autosomal genes (e.g., Figure 3). We do find some support for the faster-X evolution of male expression levels among genes in repressive chromatin that are expressed narrowly in male reproductive tissues, but the evidence is not exceedingly strong (Figure S18). Most notably, we fail to detect the faster-X effect when we limit the analysis to genes in repressive chromatin that are narrowly expressed in female reproductive tissues (Figure S18), consistent with our earlier analysis of chromosome-wide correlations of expression (Figure 3). Therefore, genes with limited expression in females do not experience faster-X expression evolution.

### Faster-X expression evolution is not driven by relaxed constraints

Accelerated evolutionary divergence can be the result of relaxed selective constraints or an elevated rate of adaptive evolution. To distinguish between these two explanations for increased divergence in gene expression one can examine intraspecific variation in expression levels [57], [71], [72]. If relaxed selective constraints were responsible for greater divergence, we would expect increased intraspecific variation in genes with rapidly evolving expression levels. Conversely, if the fast evolution of gene expression is driven by positive selection, we expect rapidly evolving genes to have equivalent (or less) expression polymorphism when compared to non-rapidly evolving genes. In making these interpretations we assume that expression variation segregating in natural populations has neutral or slightly deleterious fitness effects, an assumption common to the interpretation of DNA sequence polymorphism and divergence data [73], [74].

**Figure 9:**
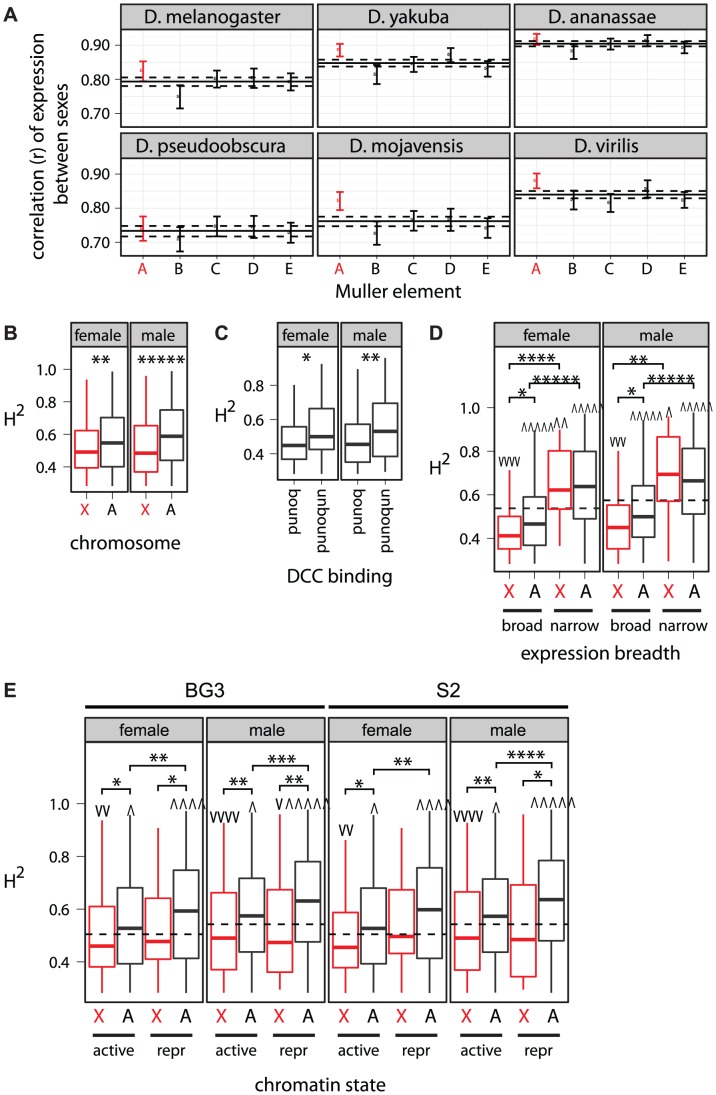
Intraspecific expression variation, X-linkage, DCC binding, expression breadth, and chromatin state. (A) Pairwise correlations of gene expression between the sexes are shown for genes on each chromosome arm, using expression measurements from six species. Correlations are represented as in Figure 1. (B–E) Boxplots show the distribution of 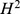 of expression level in *D. melanogaster* females or males for genes with significant heritability. Horizontal dashed lines show the genome-wide average 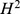. Asterisks indicate significant differences between subsets of genes. Groups of genes whose 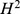 is greater 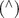 or less (v) than the rest of the genome are marked. Mann-Whitney U tests were used to assess significant differences (one symbol = 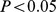, two symbols = 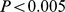, three symbols = 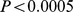, four symbols = 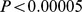, five symbols = 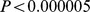). (B) Genes were divided into those that are X-linked (X, red) and those that are autosomal (A). (C) X-linked genes were divided into those that are bound by the DCC and those that are not. (D–E) Genes were further divided into (D) those that are broadly or narrowly expressed and (E) those that are in transcriptionally active or repressive (repr) chromatin measured in either BG3 or S2 cells.

One way to estimate intraspecific variation is to compare expression levels between females and males of the same species. Higher correlation between sexes suggests greater constraint on gene expression. We find that X-linked expression levels are often more correlated between the sexes than autosomal expression levels (Figure 9A), suggesting that X-linked expression levels are not under relaxed constraints.

We also used available calculations of the broad sense heritability (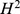) of gene expression measured in whole flies from 40 inbred *D. melanogaster* lines [75] as an estimate of the intraspecific variation in gene expression contributed by genetic factors. Higher 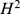 implies greater genetic variation underlying gene expression levels, which suggests relaxed selective constraints. In the results presented below, all genes with estimates of 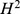 were included, but we obtain similar results if we limit ourselves to only genes included in our analysis of expression divergence. Consistent with a previous analysis of the same data [75] and independent experiments in *Drosophila simulans* [76], we detect significantly reduced 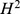 among X-linked genes (Figure 9B). This provides further evidence that the expression regulation of X-linked genes is not under relaxed selective constraints and that the faster-X effect is not a result of relaxed constraints.

The faster-X evolution of expression is most pronounced for genes that are unbound by the DCC, in transcriptionally repressive chromatin, or narrowly expressed (Figure 5A, Figure 6, Figure 8). If the faster-X effect were the result of relaxed selective constraints, we should observe increased 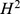 values in genes with the most pronounced faster-X effect. Consistent with this prediction, X-linked genes that are unbound by the DCC have higher 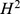 than bound genes (Figure 9C), suggesting that unbound genes are under relaxed constraints. Comparisons between X-linked and autosomal genes with similar expression breadth or in similar chromatin environments, however, suggest that the faster-X effect is not the result of relaxed constraints. For example, while narrowly expressed genes have elevated 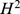 values, there is not a significant difference in 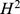 between X-linked and autosomal narrowly expressed genes (Figure 9D). Additionally, X-linked genes in repressive chromatin tend to have lower 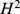 than autosomal genes in repressive chromatin (Figure 9E). Faster-X expression evolution is therefore unlikely to be a result of relaxed selective constraints on X-linked expression levels.

## Discussion

We showed that X-linked gene expression levels in *Drosophila* have more interspecific divergence than autosomal expression levels (Figure 1, Figure 2, Figure 4), demonstrating faster-X evolution of gene expression in this genus. A similar faster-X effect has been observed for expression levels in *Drosophila* embryos (Kayserili, Gerrard, Tomancak, and Kalinka, in review, http://arxiv.org/abs/1209.0968). The faster-X effect is most pronounced for genes that are unbound by the DCC (Figure 5), in transcriptionally repressive chromatin (Figure 6), and narrowly expressed in a limited number of tissues (Figure 8), as summarized in Table 1. The expression levels of X-linked genes are not more variable within species (Figure 9), suggesting that the faster-X evolution of expression is not the result of relaxed selective constraints. We therefore hypothesize that the faster-X effect is driven by a higher rate of adaptive substitutions that affect the expression of X-linked genes relative to those that affect autosomal gene expression levels. Below, we expand on this hypothesis, explaining how allelic dominance, dosage compensation, and population size may contribute to the faster-X evolution of gene expression in *Drosophila*.

**Table 1:** Factors that contribute to the faster-X evolution of gene expression.

Factor	Effect
DCC binding	X-linked genes unbound by the DCC have faster evolving expression levels
Chromatin environment	Faster-X effect most pronounced for genes in transcriptionally repressive chromatin
Expression breadth	Faster-X effect most pronounced for narrowly expressed genes in repressive chromatin
Sex-biased expression	Faster-X evolution of genes with biased expression in males and expression in both sexes
	No faster-X effect for genes with biased expression in females

### Adaptive evolution of DCC proteins and HASs

Our analysis relies on inferences of DCC binding and HASs based on experiments performed in *D. melanogaster* cells [43], [45]. DCC proteins have experienced adaptive evolution along the *D. melanogaster* lineage [27], [77], as have three HASs on the X chromosome [78]. Despite the potential for enhanced expression divergence because of this rapid evolution, we see greater conservation of expression levels associated with DCC bound genes (Figure 5A). This result implies that, despite the accelerated evolution of DCC components and their binding sites, DCC binding is likely to be conserved across species. Furthermore, if DCC binding sites are turning over, this makes our discovery of a relationship between DCC binding and expression divergence conservative.

### Chromatin environment, expression breadth, and the faster-X effect

DCC binding and chromatin states were inferred in a limited number of *D. melanogaster* cell lines. Genes that were never identified as bound by the DCC are either never compensated (because their dose does not need to be tightly controlled) or are compensated in cell types other than those studied thus far. Similarly, genes categorized as in regions of transcriptionally repressive chromatin are likely to be transcriptionally activated in a tissue-specific manner that differs from their state in S2 or BG3 cells. In this way, chromatin state can be used as a second, independent measurement of expression breadth: genes inferred to be in repressive chromatin can be assumed to be narrowly expressed, while genes in active chromatin are likely to be broadly expressed (Figure 7C).

Narrowly expressed genes tend to have rapidly evolving protein coding sequences, possibly because they are under fewer evolutionary constraints [19], [66]. Not only can these relaxed constraints permit faster evolution by purely neutral processes, but genes that are under fewer constraints are also expected to have a higher likelihood of adaptive fixations because they have less pleiotropic restrictions on their evolution [79]. Similarly, genes that are unbound by the DCC, genes that are in transcriptionally repressive chromatin, and narrowly expressed genes have rapidly evolving expression levels (Figure 5, Figure 6, Figure 7). In addition, these genes also have more intraspecific variation in expression (Figure 9), as do genes with fewer annotated functions [80], suggesting that the regulation of their expression is under relaxed constraints. If the faster-X evolution of gene expression is driven by positive selection, the faster-X effect should be most pronounced in genes that are most likely to experience adaptive substitutions. Genes in transcriptionally repressive chromatin and narrowly expressed genes do indeed have the most robust evidence for a faster-X effect (Figure 6, Figure 8), supporting an adaptive model of faster-X expression evolution.

### A framework for adaptation-driven faster-X evolution of gene expression

The canonical model of faster-X evolution driven by positive selection posits that X-linked recessive beneficial mutations will be exposed to selection in hemizygous males, this will lead to an increased probability of invasion for X-linked beneficial alleles, and there will be a higher rate of adaptive evolution in X-linked genes [1], [3]. Before we can apply this model to the faster-X evolution of gene expression, we must determine if two assumptions are met: 1) mutations that affect the expression of X-linked genes are themselves X-linked; 2) mutations that affect expression level are recessive. We consider each of these assumptions below, and then we develop a conceptual framework to explain the faster-X evolution of expression.

Gene expression is inherited in a polygenic manner [81], and both *cis* and *trans* acting factors are responsible for expression differences between *Drosophila* species [82], [83]. The expression divergence of X-linked genes is therefore determined by substitutions in X-linked *cis* regulatory sequences and the *trans* acting proteins that bind to them. While the *cis* regulatory elements are all X-linked, the *trans* factors can be encoded by X-linked or autosomal genes. There are *trans* factors that preferentially affect the expression of X-linked genes (e.g., some nuclear pore proteins [84], [85], the DCC [35], and chromatin modifications that are enriched on the X chromosome [64]), but these are unlikely to be the norm [65]. In addition, the preferential targeting of certain *trans* factors to X-linked loci is ultimately attributable to sequences that are enriched on the X chromosome—either X-linked motifs direct *trans* factors to the X chromosome [43], [44] or *trans* factors are attracted by other proteins that are enriched on the X chromosome because they themselves were directed there by X-linked sequences [85]. Similarly, the expression of autosomal genes is determined by X-linked and autosomal *trans* acting factors, but the *cis* regulatory sequences are all autosomal. It is well documented that *cis* regulatory changes play an important role in gene expression divergence [82], [83], [86]–[88]. Therefore, expression changes in X-linked genes are more likely to be the result of mutations in X-linked loci when compared to similar expression changes in autosomal genes. This supports the hypothesis that the faster-X evolution of gene expression is the result of an increased rate of X-linked substitutions affecting expression levels.

If the faster-X evolution of expression is driven by adaptive substitutions in the *cis* regulatory sequences of X-linked genes, we would expect to detect signatures of positive selection near genes on the X chromosome. X-linked loci in *D. melanogaster* do tend to have reduced genetic variation, and this can be attributed to genetic hitchhiking because of selection at loci within or near X-linked genes [13], [89], [90]. In addition, DNA sequence variation is positively correlated with intraspecific expression variation in *D. simulans* [76], and sequence divergence upstream of coding sequences is correlated with expression divergence between *D. melanogaster* and a close relative [83]. These patterns further support the hypothesis that the faster-X evolution of gene expression is driven by X-linked substitutions that affect expression level in *cis*.

While it is clear how X-linked mutations can affect the expression of X-linked genes, it is not obvious why those mutations would be recessive. Non-additive inheritance of gene expression levels is common [91], [92], but *cis* regulatory differences between species are more likely to be inherited in an additive manner [83], [93]. These results suggest that the phenotypic effects of mutations that affect expression in *cis* are not likely to be recessive, but what is more important is whether the fitness effects of the mutations are recessive. It is reasonable to assume that the fitness landscape near an optimum is concave, which implies that mutations that push the expression level of a gene toward the optimum will be dominant [94], [95]. Therefore, empirical results and theoretical predictions appear to challenge the assumption that beneficial mutations that affect expression in *cis* will be recessive.

**Figure 10:**
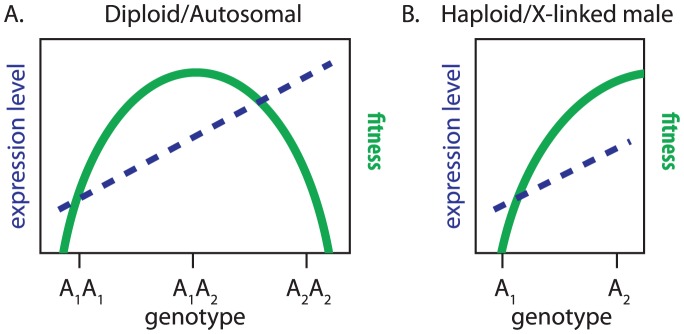
Additive phenotypic effects, fitness landscapes, and X-linkage. Expression level of a gene (dashed blue line) and relative fitness (solid green curve) are plotted for genotypes at a locus with two alleles (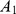 and 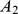). The alleles have additive phenotypic effects, with each copy of 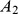 increasing expression level. (A) The locus is autosomal with phenotypic and fitness measurements collected in females or males, or it is X-linked with measurements collected in females. (B) The locus is X-linked and not dosage compensated with measurements collected in males.

Recently, however, it has been demonstrated that beneficial mutations with additive phenotypic effects can increase fitness in heterozygotes while at the same time being less fit when homozygous because they overshoot the adaptive peak [96], [97] (Figure 10A). Therefore, mutations with additive phenotypic effects can have over-dominant fitness effects as a consequence of diploidy [97]. While this could impede adaptation at autosomal loci, the dynamics of this process are likely to differ at X-linked genes because they are effectively haploid in males in the absence of dosage compensation (Figure 10B). Notably, we detect the faster-X effect in genes that appear not be dosage compensation (Figure 5). Beneficial mutations with additive phenotypic effects on the expression of these uncompensated X-linked genes may therefore be more likely to fix because selection in males does not run the risk of overshooting the fitness optimum as a consequence of diploidy (Figure 10). Further theoretical work is needed to determine whether this intuitive prediction is a feasible adaptive explanation for the faster-X evolution of gene expression.

If the aforementioned model could explain the faster-X evolution of gene expression, we would expect the faster-X effect to be limited to genes expressed in males because they would be present in the hemizygous (i.e., haploid) state. Consistent with this hypothesis, the expression levels of X-linked genes transcribed primarily in female-limited tissues do not evolve faster than autosomal genes with equivalent expression profiles (Figure 3). We do, however, detect the faster-X effect when expression is measured in either males or females (Figure 1, Figure 2, Figure 4, Figure 6, Figure 8). This is because male and female phenotypes are correlated so that selection on expression levels in males will affect the expression levels of genes that are also expressed in females [98], [99].

### Lineage-specific patterns of faster-X expression evolution in *Drosophila*

Within the *Drosophila* genus, we observe the most pronounced faster-X effect along the lineage leading to *D. ananassae* (Figure 4, Figure 6, Figure 8). The Painting of fourth (POF) protein localizes specifically to the diminutive *D. melanogaster* fourth chromosome (Muller element F, or dot chromosome) [100]. Numerous lines of evidence suggest that POF promotes the transcriptional output of genes on the fourth chromosome by an unknown RNA-binding mechanism [101]–[103]. Intriguingly, while POF is dot-chromosome-specific in most *Drosophila* species, it also co-localizes with the DCC on the X chromosome in *D. ananassae* males [104].

POF localization to the *D. ananassae* X chromosome in males suggests that X-linked gene expression is uniquely affected in *D. ananassae*. This could contribute to the increased expression divergence of the X chromosome along the *D. ananassae* lineage either by directly affecting the expression levels of *D. ananassae* genes or by creating unique selection pressures on X-linked gene expression. We detect a substantial faster-X effect in female expression along the *D. ananassae* lineage (Figure 4A), despite the fact that POF does not localize to the X chromosome in *D. ananassae* females [104]. Therefore, the accentuated faster-X effect along the lineage leading to *D. ananassae* is unlikely to be a direct result of POF modifying expression levels. It is instead more likely that POF localization to the X chromosome in *D. ananassae* creates novel selection pressures on X-linked expression levels, which leads to a more pronounced faster-X effect.

### Faster-X expression evolution in *Drosophila* and mammals

Expression levels of X-linked genes diverge faster than those of autosomal genes along both internal and terminal branches of the *Drosophila* phylogeny (Figure 4). The faster-X effect in mammals, on the other hand, is limited to only some deep lineages [28]. If our conceptual framework for understanding the faster-X evolution of gene expression is correct, we should be able to use it to explain differences in the faster-X effect between *Drosophila* and mammals. We consider four hypotheses that could explain the extent of faster-X expression evolution in the two taxa, and we examine how each could contribute to the observed incongruities.

First, X chromosome gene content differs between *Drosophila* and mammals [52], [105]. These differences, however, are unlikely to be responsible for the differences in the faster-X effect between mammals and *Drosophila*. The mammalian X chromosome harbors an excess of narrowly expressed genes [52], [106], i.e., the same type of genes with the most pronounced faster-X effect in *Drosophila* (Figure 8). Therefore, we would expect an even more substantial faster-X effect in mammals if differences in X chromosome gene content were an important contributor to taxon-specific faster-X expression evolution.

Second, *Drosophila* and mammals deal with the haploid dose of the male X chromosome in different ways [29], [37]. Faster-X gene expression evolution in *Drosophila* is most pronounced for genes that are unbound by the DCC (Figure 5), and we hypothesize that the effective haploidy of X-linked alleles in uncompensated *Drosophila* genes promotes the faster-X effect (Figure 10). Mammalian dosage compensation, on the other hand, is thought to be a two step process: X chromosome gene expression is upregulated in both sexes, followed by random silencing of one X chromosome in females [29]–[31]. The specific mechanisms of mammalian X chromosome upregulation are not understood, and the phenomenon itself remains controversial [32]. Regardless of the details of mammalian dosage compensation, if the allelic dominance of fitness effects for mutations that change gene expression are affected by the mechanism of dosage compensation, the differences in dosage compensation between *Drosophila* and mammals could be responsible for the taxon-specific patterns of faster-X expression evolution [1], [3].

Third, the rate of evolution depends on mutational input and the fixation rate of those mutations. A higher autosomal mutation rate could therefore counteract a higher fixation rate on the X chromosome [107]. The mutation rate in the germline of many mammals is higher in males than females (“male mutation bias”) [4]–[8]. Because the X chromosome is transmitted through females 2/3 of the time, the population mutation rate is lower for the X chromosome than the autosomes in species with male mutation bias. This downward biased mutation rate of the X chromosome in some mammalian lineages could therefore be responsible for the lineage-specificity of the faster-X effect in mammals [107], [108].

Fourth, if the faster-X evolution of gene expression is driven by adaptive substitutions, as we propose, it is likely to be sensitive to 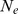 [9], [10]. In small populations a larger fraction of mutations will be effectively neutral [109], which will decrease the number of beneficial mutations fixed by positive selection. The higher 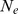 of *Drosophila* relative to mammals may therefore be more permissive of adaptive faster-X evolution [10].

In summary, we conclude that the difference in the extent of the faster-X evolution of gene expression between *Drosophila* and mammals could be a result of the unique mechanism of dosage compensation in *Drosophila*, the pervasiveness of male mutation bias, and/or the differences in 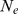 between taxa. Determining which factor is most important will require additional theoretical and empirical work to identify the key determinants of gene expression evolution, the nature of selection on expression, and the effects of gene dosage on the dominance of fitness effects.

## Methods

### Expression measurements

We obtained microarray measurements of expression from whole fly or head from previously published results [50]–[52]. We calculated the expression level of each gene by first taking the median signal across all probes for each gene within each replicate, and then calculating the median for each gene across all replicates. As an alternative approach, we used expression levels estimated in the LIMMA package of Bioconductor [110], as described previously [52].

We tested for significant differences in expression between males and females (i.e., sex-biased expression) using moderated t-tests implemented in the LIMMA package with the empirical Bayes function to pool sample variances toward a common value [110], as described previously [52]. We corrected for multiple tests using a FDR [111] of 0.05 when only sex-biased expression in *D. melanogaster* was considered, and with a FDR of 0.20 when sex-biased expression in all species was considered. Genes with significantly higher expression in males were classified as having male-biased expression, and those with higher expression in females as female-biased.

RNA-seq data collected from whole *D. melanogaster*, *D. pseudoobscura*, and *D. mojavensis* males and female or *D. melanogaster*, *D. pseudoobscura*, *D. willistoni*, and *D. mojavensis* heads were obtained from previously published results [52]. Reads longer than 36 bases (bp) were trimmed to 36 bp so that all reads were the same length, and reads were then mapped to the transcriptome using BWA [112]. Any read mapping to multiple locations in the genome was discarded, and genes with fewer than 50 mapped reads were excluded from the subsequent analysis. The expression level of each gene was estimated as the number of unique reads mapping to the gene standardized by the total number of mapped reads and the transcript length.

We extracted only those genes present as 1∶1 orthologs on the same chromosome arm in all species under consideration, and we quantile normalized the expression levels so that they are identically distributed across all species. We next calculated SpearmanÕs 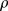 between all pairwise comparisons of expression levels from the species under consideration. This was repeated for each chromosome arm. Confidence intervals (CIs) of 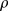 were estimated by bootstrapping the data 1000 times in the R statistical computing environment [113]. We also calculated correlations of expression between sexes within each species.

Microarray measurements of expression were obtained for 14 adult *D. melanogaster* tissues from FlyAtlas [58], and the expression breadth was determined for each gene as described previously [19]. Briefly, we calculated 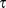 [114], a metric that ranges between 0 (for broadly expressed genes) to 1 (for narrowly expressed genes). Genes were said to be narrowly expressed in a tissue if 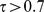, and genes with 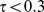 were classified as broadly expressed.

### Phylogenetic analysis

We used 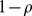 as an estimate of the pairwise divergence in expression between *D. melanogaster*, *D. yakuba*, *D. ananassae*, *D. pseudoobscura*, *D. mojavensis*, and *D. virilis*. We then reconstructed the phylogenetic relationships using the method of Fitch and Margoliash [115] implemented in the PHYLIP software package [116]. Bootstrap support for phylogenetic nodes was estimated by resampling the 1∶1∶1∶1∶1∶1 orthologs 1000 times. Branch lengths were estimated using the method of Fitch and Margoliash [115] with a fixed tree topology implemented in the PHYLIP software package [116]. CIs of the branch lengths were calculated by bootstrapping the data 1000 times. Bootstrap support and branch lengths were estimated for all 1∶1∶1∶1∶1∶1 orthologs, and this was repeated for genes on each chromosome arm separately. All bootstrapping was performed using the R statistical computing environment [113].

### DCC binding

We obtained a list of genes bound by the DCC identified using ChIP-chip in three cell types: SL2 embryonic cell culture, larval wing imaginal disc cell culture, and late embryo [45]. A gene was said to be bound by the DCC if it is bound in at least one cell type. In addition, a second list of DCC bound genes was kindly provided by D. Bachtrog [48]. Our results are robust to the gene list used in our analysis. X-linked regions identified as HASs were obtained from previously published results [43].

### Gene-wise expression divergence and distance to nearest HAS

We calculated the gene-wise expression divergence between 1∶1∶1 orthologs in *D. melanogaster*, *D. yakuba*, and *D. ananassae* as:
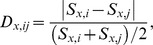
where 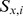 and 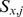 are the expression levels of ortholog 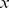 in species 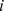 and 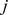, respectively. We then calculated Spearman's 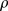 between 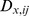, 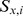, and distance to the nearest HAS (where 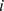 = *D. melanogaster*). From these pairwise correlations, we calculated partial correlations to determine the direct relationship of each pair of values [117]. The CIs of the pairwise and partial correlations were estimated by bootstrapping the 1∶1∶1 orthologs 1000 times in the R statistical programming environment [113].

### Chromatin states

Kharchenko et al. [65] analyzed ChIP-chip results for multiple histone modifications and DNA binding proteins in two *D. melanogaster* cell lines (S2 and BG3), and they used a hidden Markov model to assign each region of the genome to one of nine chromatin states. States 1–5 are associated with transcriptionally active chromatin marks and states 6–9 with repressive marks. We used the overlap of these regions with annotated protein coding genes to determine whether each *D. melanogaster* gene is associated with a region of active or repressive chromatin marks. A gene was considered to be in active chromatin if 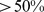 of the gene body overlaps with regions identified as containing active marks, and, conversely, a gene was considered to be in repressive chromatin in 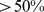 of the gene body overlaps with regions identified as harboring repressive marks. We obtain similar results when use overlap cutoffs of 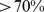 and 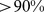.

### Intraspecific expression variation

We obtained estimates of broad sense heritability (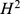) for *D. melanogaster* genes from a published analysis of microarray expression measurements in females and males from 40 inbred lines [75]. These estimates were calculated using an analysis of variance (ANOVA), and we considered only genes in which the line term (the estimate of 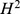) was significant at a FDR of 0.05.

## Supporting Information

Figure S1 Faster-X evolution of gene expression using an alternative analysis of microarray data. Pairwise correlations of gene expression are shown for genes on each chromosome arm, using expression measurements from females (top) or males (bottom). Expression levels were estimated using the LIMMA package of Bioconductor [110] (see Methods). See Figure 1 for a description of the graphs.
(PDF)

Figure S2 Faster-X evolution of gene expression using RNA-seq data. Pairwise correlations of gene expression are shown for genes on each chromosome arm, using expression measurements from females (top) or males (bottom) collected by RNA-seq. See Figure 1 for a description of the graphs.
(PDF)

Figure S3 Faster-X evolution of gene expression using microarray data with only the genes included in the RNA-seq data set. Pairwise correlations of gene expression are shown for genes on each chromosome arm, using expression measurements from females (top) or males (bottom) collected by microarray. Only those genes present in both the microarray and RNA-seq data sets are included. See Figure 1 for a description of the graphs.
(PDF)

Figure S4 Faster-X evolution of gene expression measured in head using RNA-seq. Pairwise correlations of gene expression are shown for genes on each chromosome arm, using expression measurements from female (top) or male (bottom) heads collected with RNA-seq. See Figure 1 for a description of the graphs.
(PDF)

Figure S5 Faster-X evolution of gene expression with D. melanogaster male-biased genes removed. Pairwise correlations of gene expression are shown for genes on each chromosome arm, using expression measurements from females (top) or males (bottom). Genes with male-biased expression in *D. melanogaster* were excluded. See Figure 1 for a description of the graphs.
(PDF)

Figure S6 Faster-X evolution of gene expression with male-biased genes removed. Pairwise correlations of gene expression are shown for genes on each chromosome arm, using expression measurements from females (top) or males (bottom). Genes with male-biased expression in any of the six species were excluded. See Figure 1 for a description of the graphs.
(PDF)

Figure S7 Faster-X evolution of gene expression with genes with biased expression in male reproductive tissues removed. Pairwise correlations of gene expression are shown for genes on each chromosome arm, using expression measurements from females (top) or males (bottom). Genes with biased expression in either testis or accessory gland were excluded. See Figure 1 for a description of the graphs.
(PDF)

Figure S8 New genes do not exhibit faster-X evolution of gene expression Pairwise correlations of gene expression between *D. melanogaster* and *D. yakuba* (mel-yak) and *D. melanogaster* and *D. ananassae* (mel-ana) for genes on each chromosome arm. Correlations were calculated using genes shared by all species in the *Drosophila* genus and genes that arose along the lineage leading to *D. melanogaster* after the split with the *Drosophila* subgenus [61].
(PDF)

Figure S9Phylogenetic reconstruction using the correlation of expression. We reconstructed the evolutionary relationships of the six species using the pairwise correlation of expression levels in (A) females and (B) males. A distance matrix of 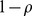 was used to build the phylogenies using the Fitch and Margoliash [115] method. We bootstrap sampled the genes 1000 times to estimate the support for each node, and the percent of bootstrap replicates supporting each node is given on the tree.(PDF)

Figure S10Expression levels of genes in transcriptionally active and repressive chromatin Boxplots show the distribution of expression levels for *D. melanogaster* genes in transcriptionally active and repressive (repress) chromatin. Horizontal dashed lines represent the genome-wide expression level. Chromatin states were measured in two cell lines (BG3 and S2), and expression levels were measured in either females or males. In addition, genes were divided into those that are autosomal and those that are X-linked. Significant differences between the expression levels of genes in active and repressive chromatin are indicated by asterisks (*** 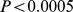, ***** 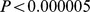).(PDF)

Figure S11 Faster expression evolution of un-dosage-compensated X-linked genes not associated with active chromatin. This graph is the same as the one in Figure 6 except that X-linked genes are now divided into those that are both bound by the DCC and in active chromatin or unbound by the DCC and in repressive chromatin.
(PDF)

Figure S12Narrowly expressed genes have greater expression divergence. Spearman's rank order correlation between 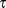 and expression divergence is plotted, along with the 95% CI of the correlation. Larger values of 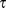 indicate narrower expression breadth. Divergence was measured between *D. melanogaster* (mel) and both *D. yakuba* (yak) and *D. ananassae* (ana) using measurements from both females and males. The dashed line indicates the null expectation of no correlation.(PDF)

Figure S13DCC bound genes tend to be broadly expressed. X-linked genes were divided into those that are bound and unbound by the DCC. (A) Expression breadth (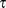) was compared between bound and unbound genes using a Mann-Whitney U test (***** 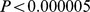). Larger values of 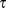 indicate narrower expression breadth. (B) SpearmanÕs rank order correlation between distance from the nearest HAS and 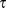 is plotted, along with the 95% CI of the correlation. The dashed line indicates the null expectation of no correlation.(PDF)

Figure S14Genes in repressive chromatin are more narrowly expressed. Genes were divided into those that are in transcriptionally active and repressive chromatin using data from two cell lines: BG3 and S2. (A) Expression breadth (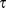) was compared between genes in active and repressive chromatin using a Mann-Whitney U test (***** 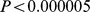). Larger values of 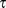 indicate narrower expression breadth. (B) Genes were additionally divided into those that are broadly and narrowly expressed, and the counts of genes in each expression breadth and chromatin state are plotted for data collected in BG3 cells (S2 data are available in Figure 7). Fisher's exact test was used to determine if there is a significantly non-random distribution of genes in the four classes.(PDF)

Figure S15 The faster-X effect is limited to narrowly expressed genes in transcriptionally repressive chromatin, with chromatin environment measured in BG3 cells. This figure is the same as Figure 8 except chromatin state was measured in BG3 cells.
(PDF)

Figure S16 The faster-X effect is limited to narrowly expressed genes in transcriptionally repressive chromatin, with chromatin environment measured in BG3 cells. This figure is the same as Figure 8 and Figure S15 except that genes with the lowest 5% expression levels were excluded.
(PDF)

Figure S17Expression breadth, chromatin environment, and X-linkage. The expression breadth (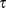) of genes in transcriptionally active and repressive chromatin on the X chromosome and autosomes is plotted. Larger values of 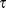 indicate narrower expression breadth. Chromatin state was inferred in BG3 and S2 cells. Significant differences between X-linked and autosomal genes in the same chromatin context are indicated by asterisks (* 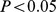, *** 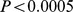, **** 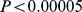). (A) Median expression breadth across the entire genome is indicated by a dashed line. (B) Genes were additionally divided into narrowly and broadly expressed.(PDF)

Figure S18No faster-X effect for genes narrowly expressed in female reproductive tissues. Boxplots show the pairwise divergence in expression between 1∶1∶1 orthologs in the *D. melanogaster* (mel) and *D. yakuba* (yak) or *D. ananassae* (ana) genomes measured in whole females and males (see Figure 6). Only genes that are narrowly expressed in non-reproductive tissues (non-repro), female reproductive tissues (female repro), or male reproductive tissues (male repro) are included. X-linked (X, red) and autosomal (A) genes were assigned to transcriptionally active and repressive chromatin based on the results of experiments in BG3 (top) and S2 (bottom) cells. The horizontal dashed line indicates the genome-wide average pairwise divergence for narrowly expressed genes. Subsets of narrowly expressed genes whose pairwise divergence is significantly different than all other narrowly expressed genes are marked (N). The same was done for subsets of narrowly expressed genes that reside in repressive chromatin (R). Significant differences between X-linked and autosomal genes in the same chromatin state and with the same expression breadth are marked with asterisks. Mann-Whitney U tests were used to assess significant differences (one symbol = 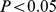, two symbols = 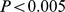, three symbols = 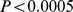, four symbols = 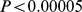, five symbols = 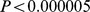).(PDF)

Table S1 DCC binding and chromatin state. Genes were called as bound by the DCC if they were bound in S2 cells (SL2); either SL2, larval wing imaginal disc, or late embryonic cells (Any); or based on a separate analysis of the data (Bachtrog) [48]. Chromatin states (chr.state) were inferred in S2 cells or BG3 cells. The number of genes in each DCC binding and chromatin state class (num_genes) were tested for independence using Fisher's exact test, and the P value of this test is reported (FET.p).
(PDF)
