# Supplementary figures and images for: Faster-X Evolution of Gene Expression in Drosophila
Source: PLoS Genet. 2012 Oct 11;8(10):e1003013. doi: 10.1371/journal.pgen.1003013 (PMC3469423; doi:10.1371/journal.pgen.1003013)

**A. Female expression levels**

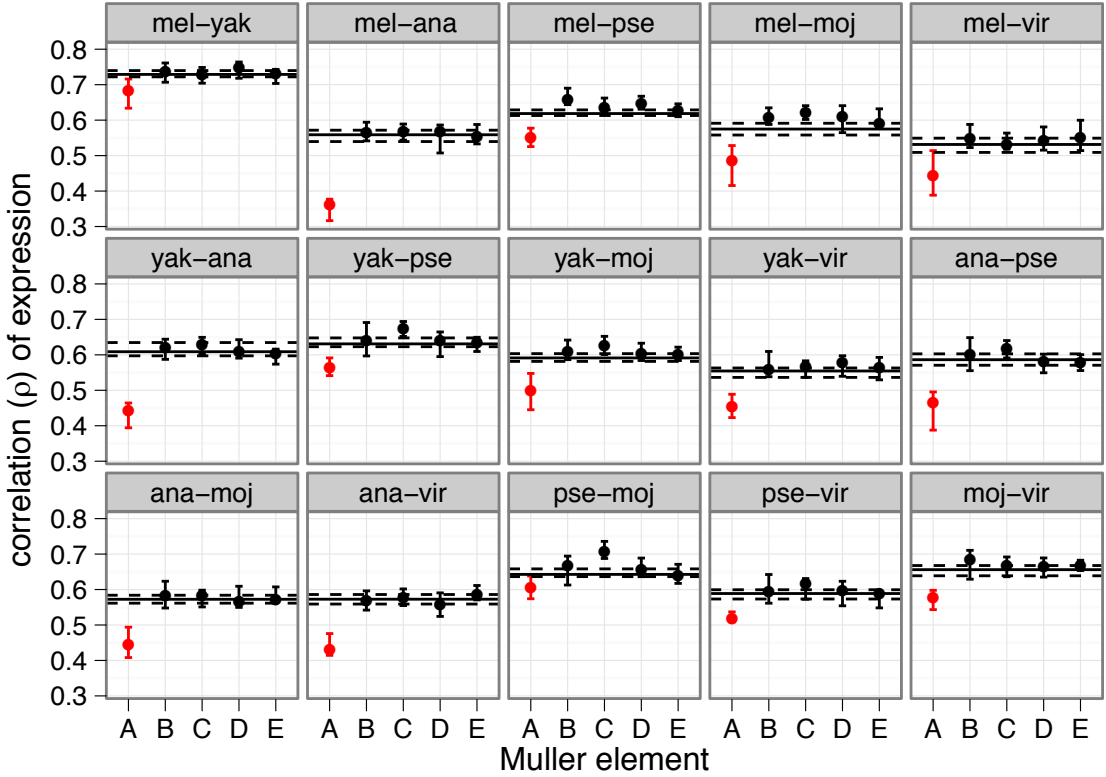

**B. Male expression levels**

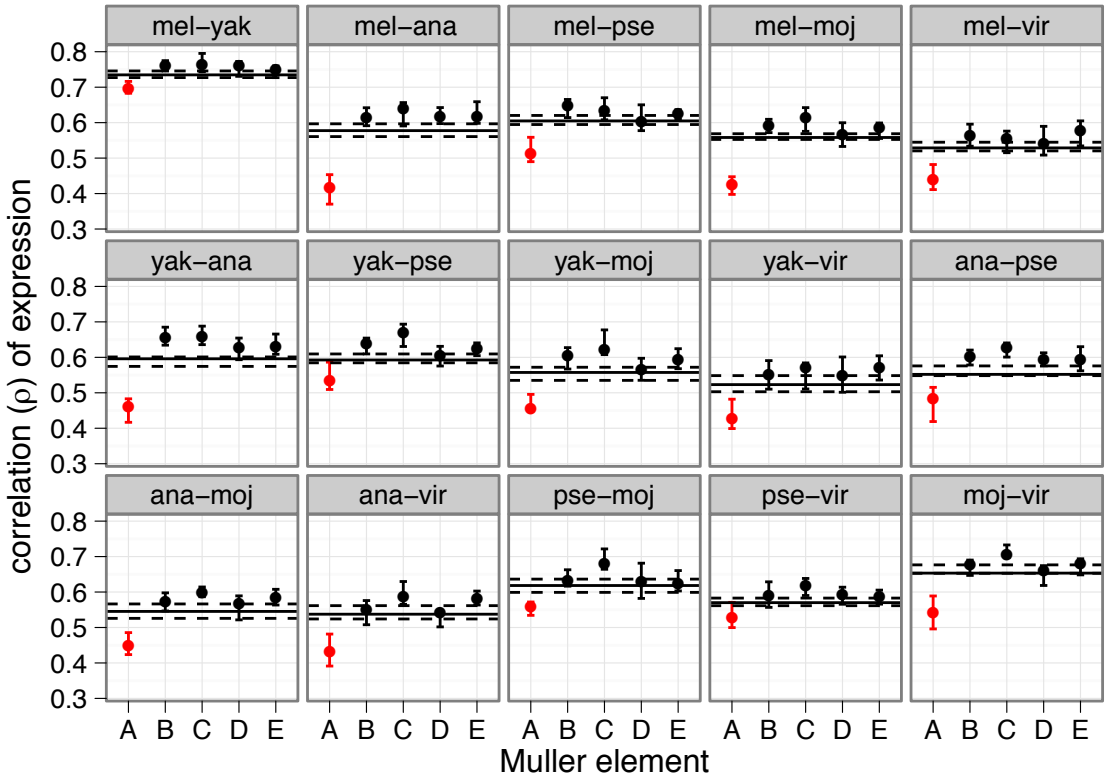

Supplement: Figure S1 — Faster-X evolution of gene expression using an alternative analysis of microarray data. Pairwise correlations of gene expression are shown for genes on each chromosome arm, using expression measurements from females (top) or males (bottom). Expression levels were estimated using the LIMMA package of Bioconductor [110] (see Methods). See Figure 1 for a description of the graphs. (PDF) [file pgen.1003013.s001.pdf]

correlation ( $\rho$ ) of expression level

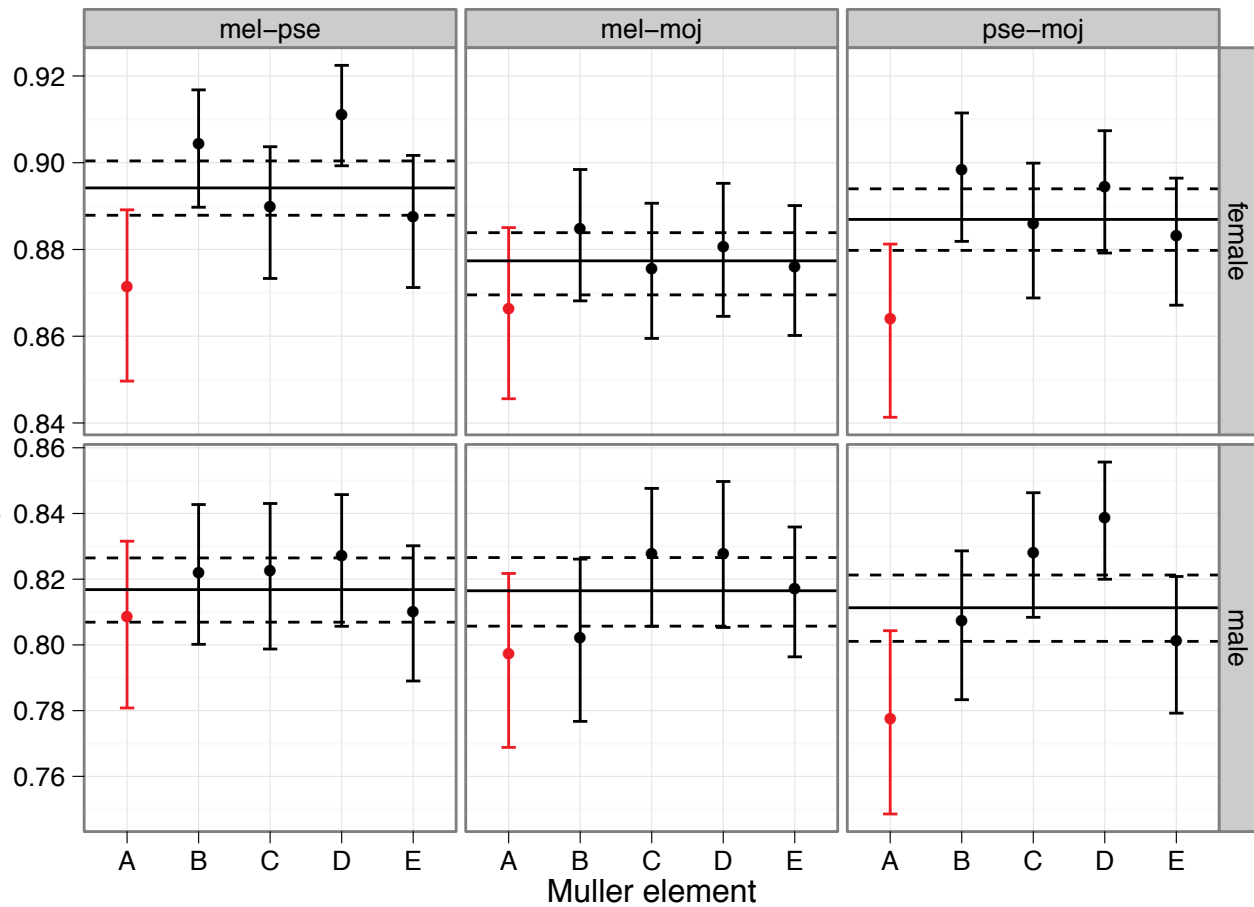

Supplement: Figure S2 — Faster-X evolution of gene expression using RNA-seq data. Pairwise correlations of gene expression are shown for genes on each chromosome arm, using expression measurements from females (top) or males (bottom) collected by RNA-seq. See Figure 1 for a description of the graphs. (PDF) [file pgen.1003013.s002.pdf]

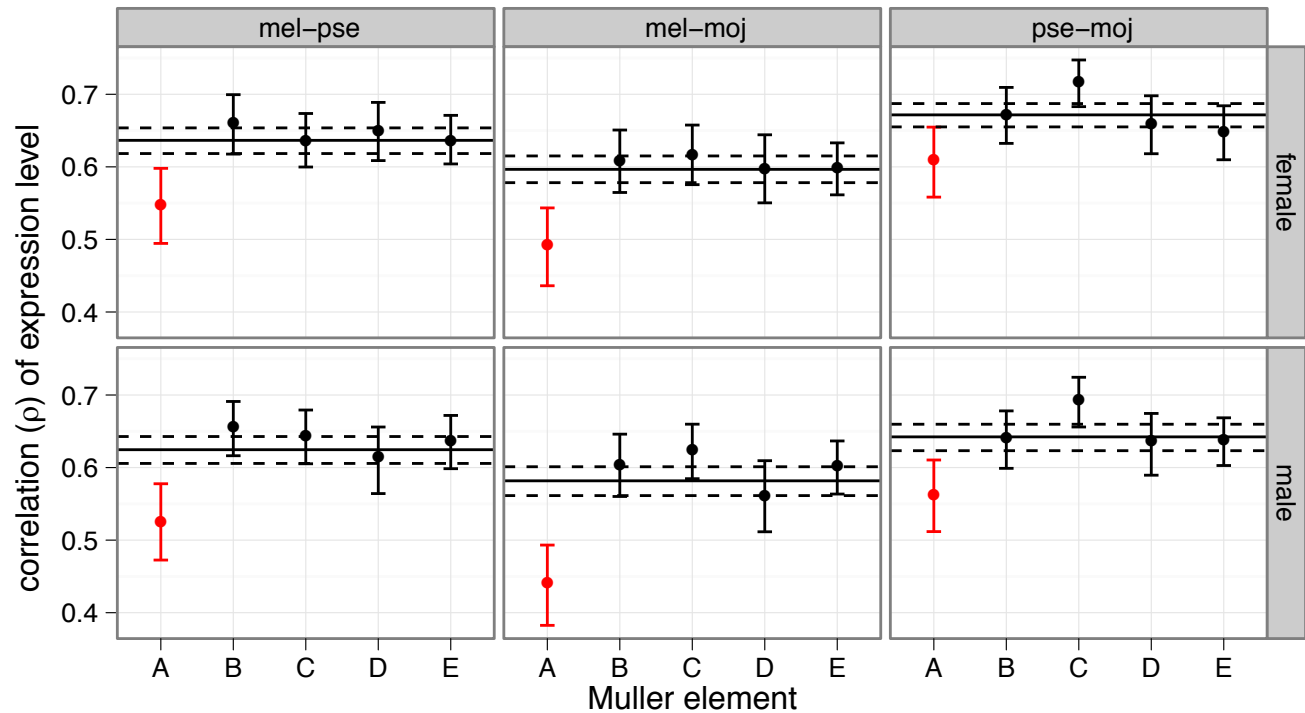

Supplement: Figure S3 — Faster-X evolution of gene expression using microarray data with only the genes included in the RNA-seq data set. Pairwise correlations of gene expression are shown for genes on each chromosome arm, using expression measurements from females (top) or males (bottom) collected by microarray. Only those genes present in both the microarray and RNA-seq data sets are included. See Figure 1 for a description of the graphs. (PDF) [file pgen.1003013.s003.pdf]

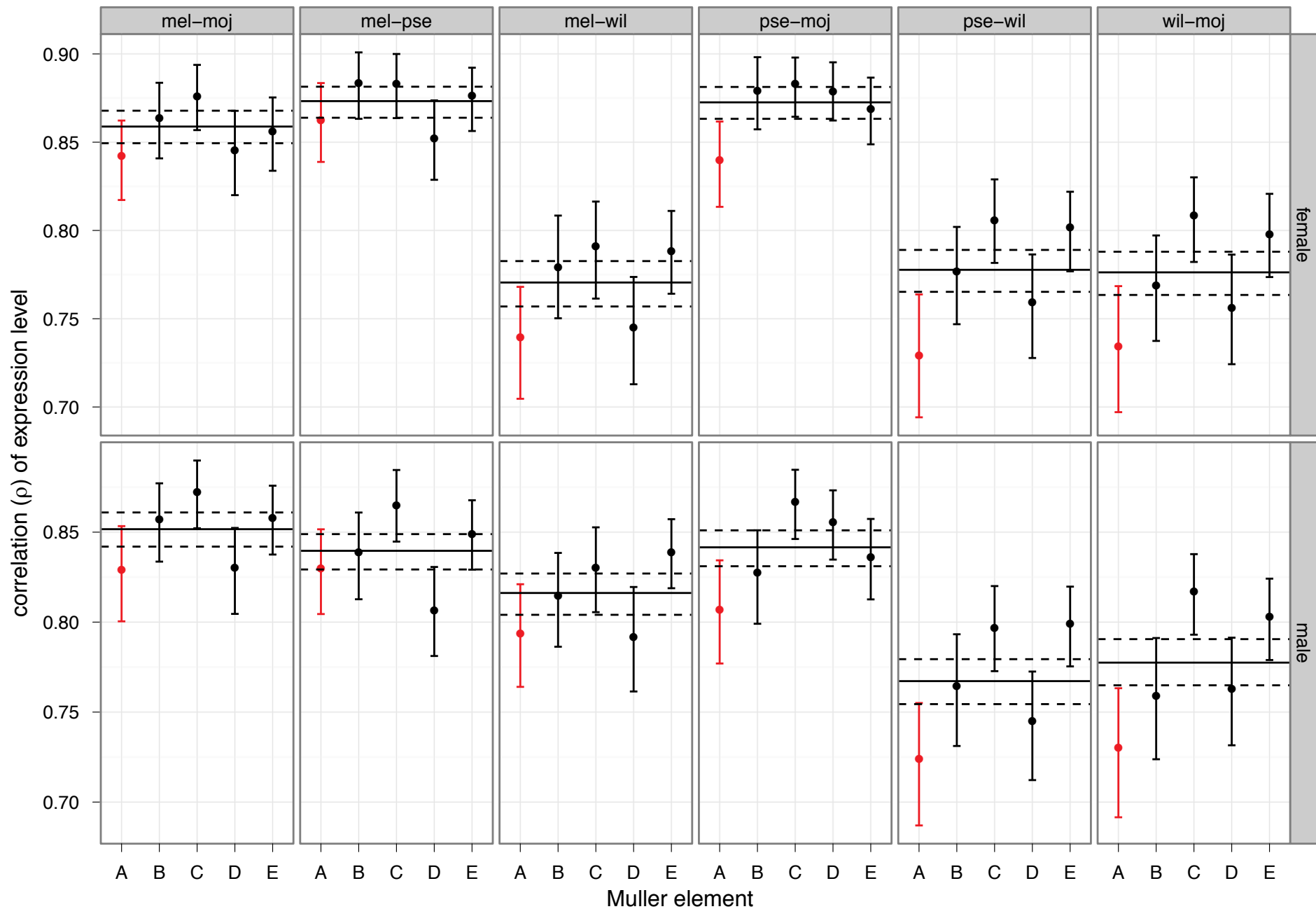

Supplement: Figure S4 — Faster-X evolution of gene expression measured in head using RNA-seq. Pairwise correlations of gene expression are shown for genes on each chromosome arm, using expression measurements from female (top) or male (bottom) heads collected with RNA-seq. See Figure 1 for a description of the graphs. (PDF) [file pgen.1003013.s004.pdf]

## female expression

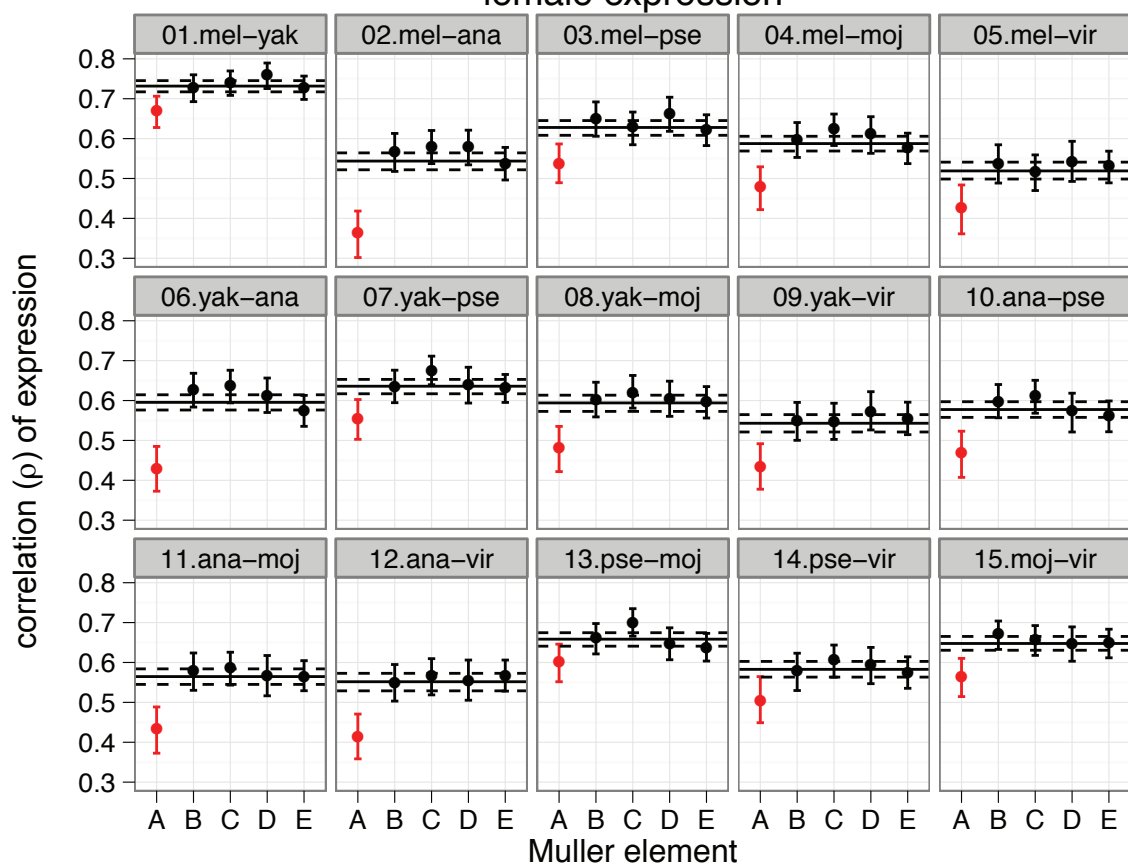

## male expression

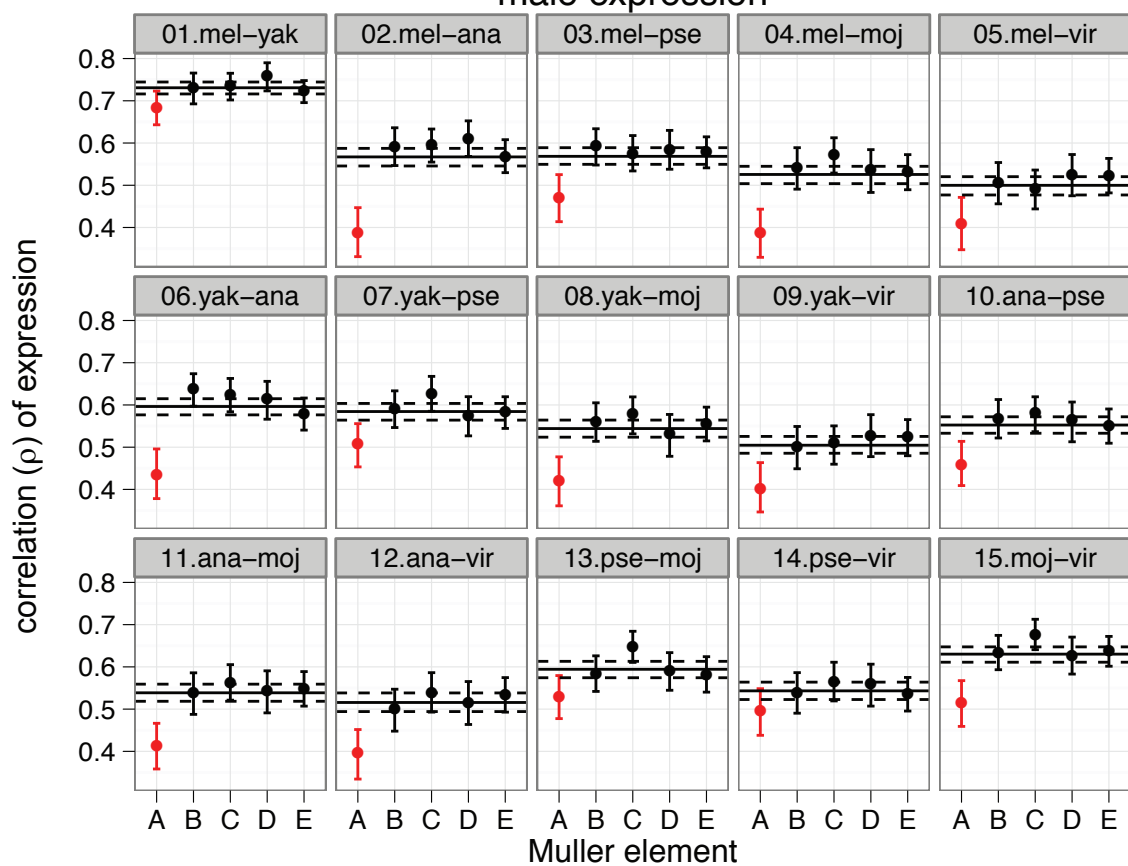

Supplement: Figure S5 — Faster-X evolution of gene expression with D. melanogaster male-biased genes removed. Pairwise correlations of gene expression are shown for genes on each chromosome arm, using expression measurements from females (top) or males (bottom). Genes with male-biased expression in D. melanogaster were excluded. See Figure 1 for a description of the graphs. (PDF) [file pgen.1003013.s005.pdf]

## female expression

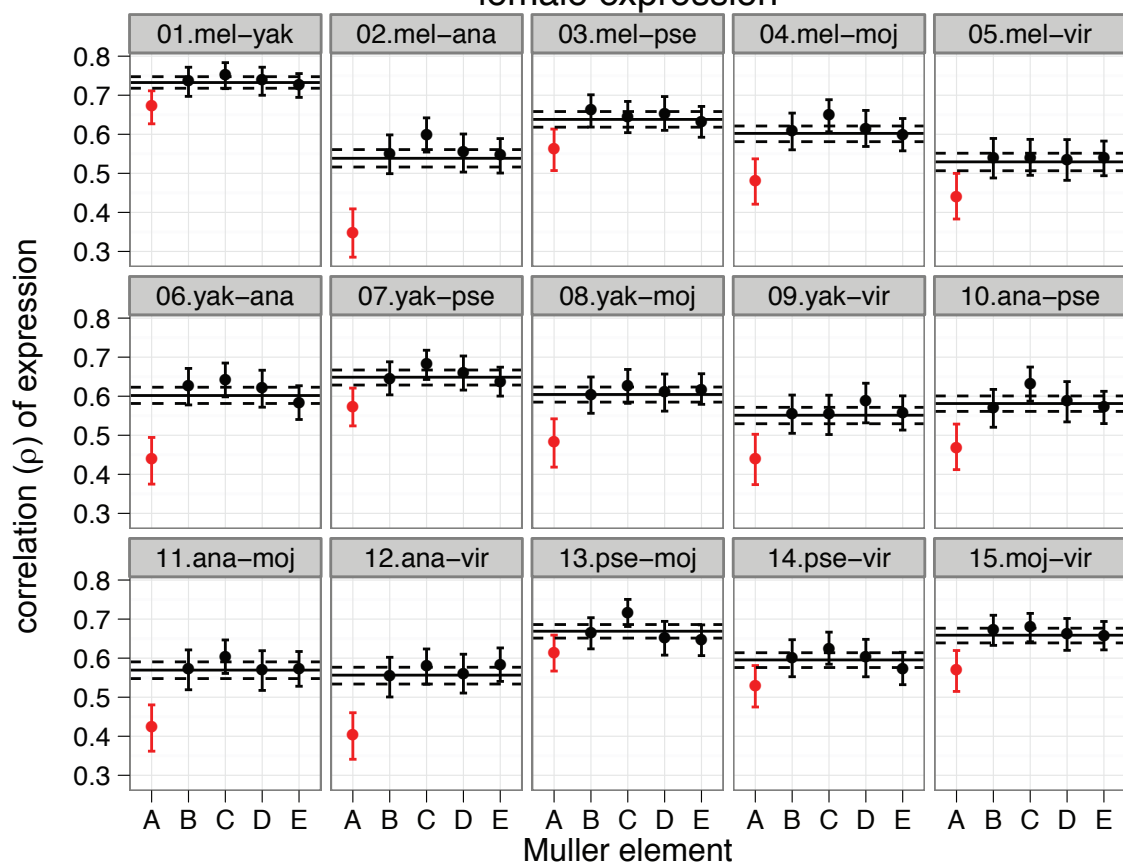

## male expression

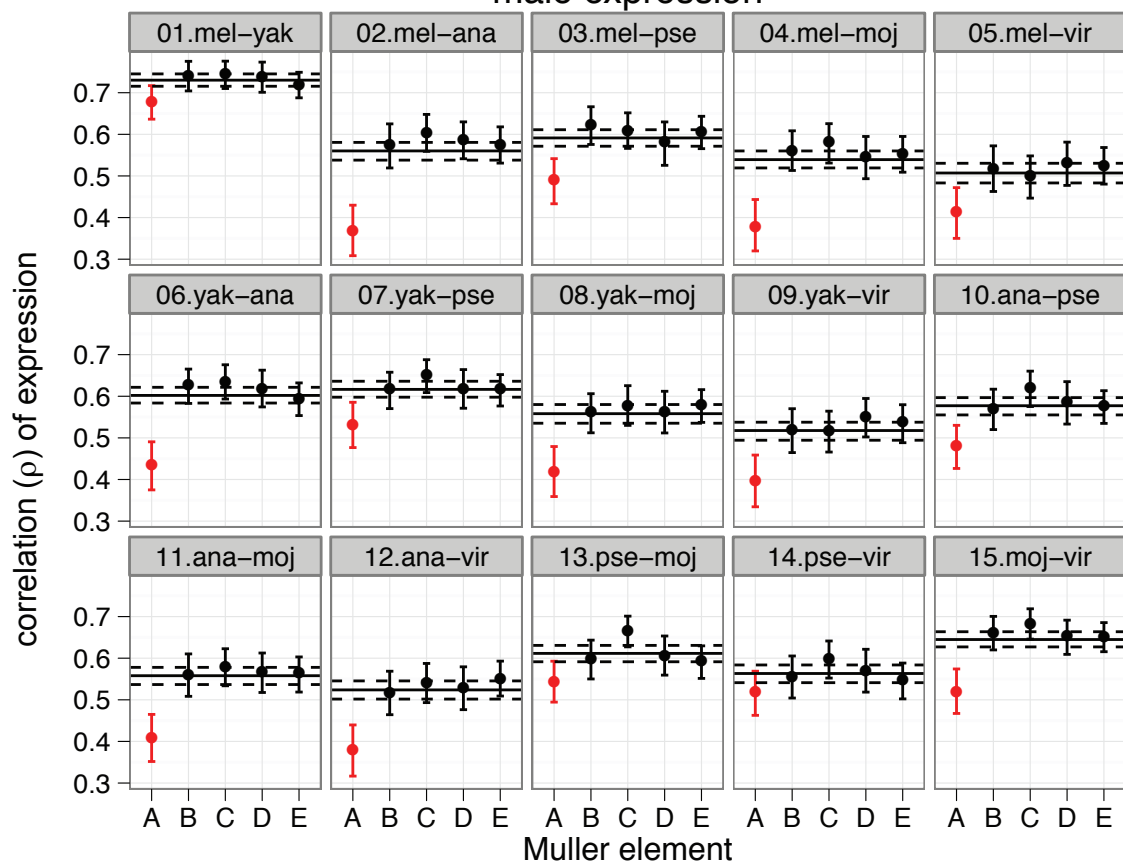

Supplement: Figure S6 — Faster-X evolution of gene expression with male-biased genes removed. Pairwise correlations of gene expression are shown for genes on each chromosome arm, using expression measurements from females (top) or males (bottom). Genes with male-biased expression in any of the six species were excluded. See Figure 1 for a description of the graphs. (PDF) [file pgen.1003013.s006.pdf]

## female expression

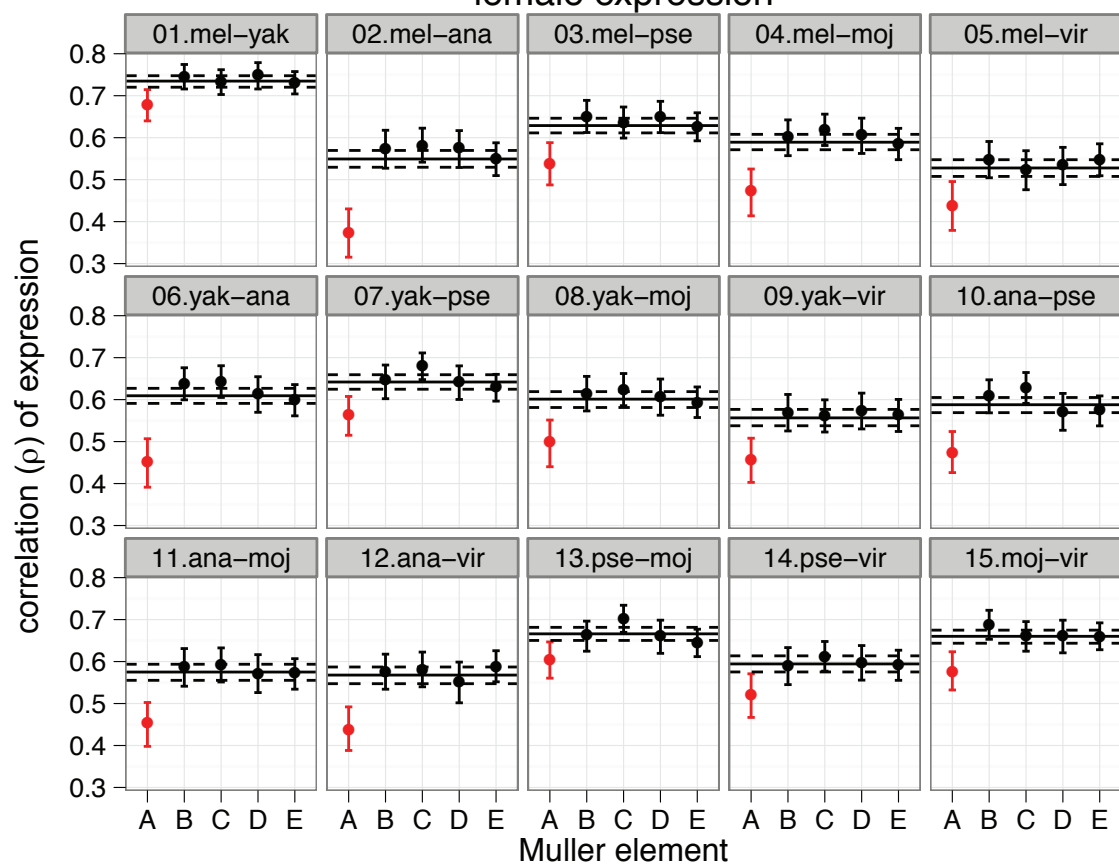

## male expression

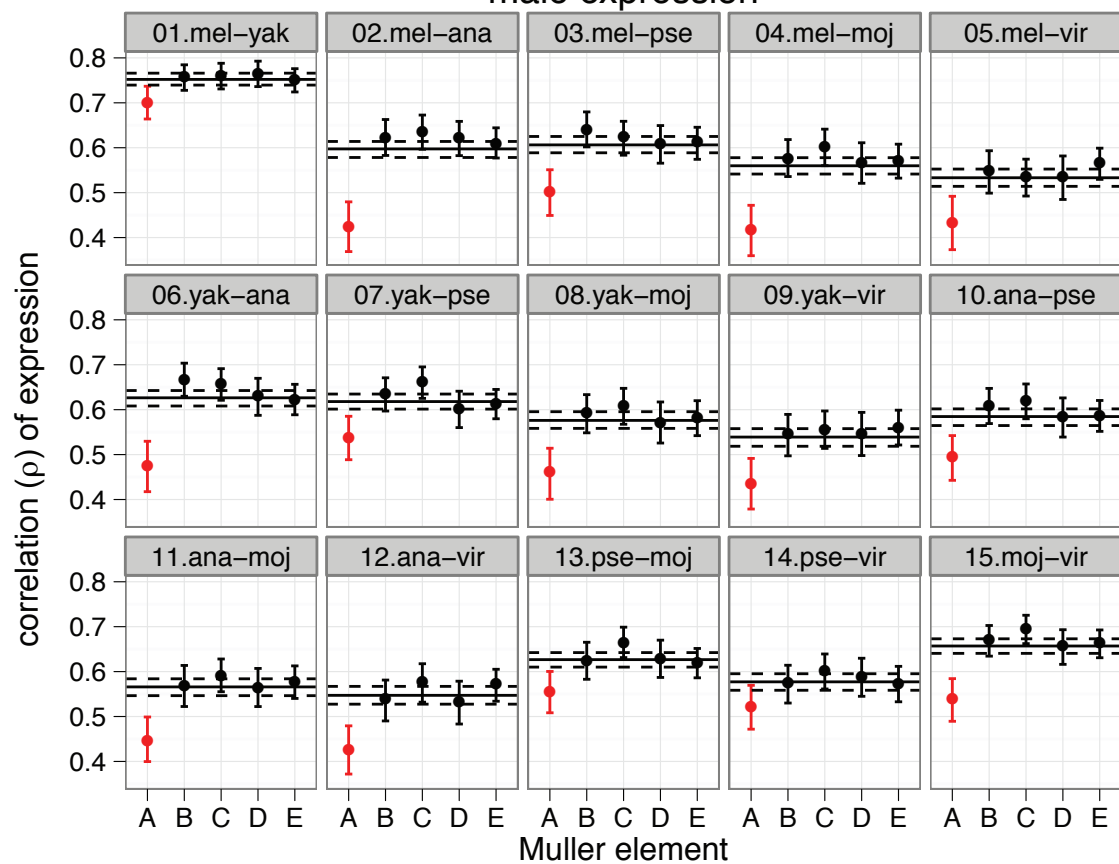

Supplement: Figure S7 — Faster-X evolution of gene expression with genes with biased expression in male reproductive tissues removed. Pairwise correlations of gene expression are shown for genes on each chromosome arm, using expression measurements from females (top) or males (bottom). Genes with biased expression in either testis or accessory gland were excluded. See Figure 1 for a description of the graphs. (PDF) [file pgen.1003013.s007.pdf]

correlation of expression

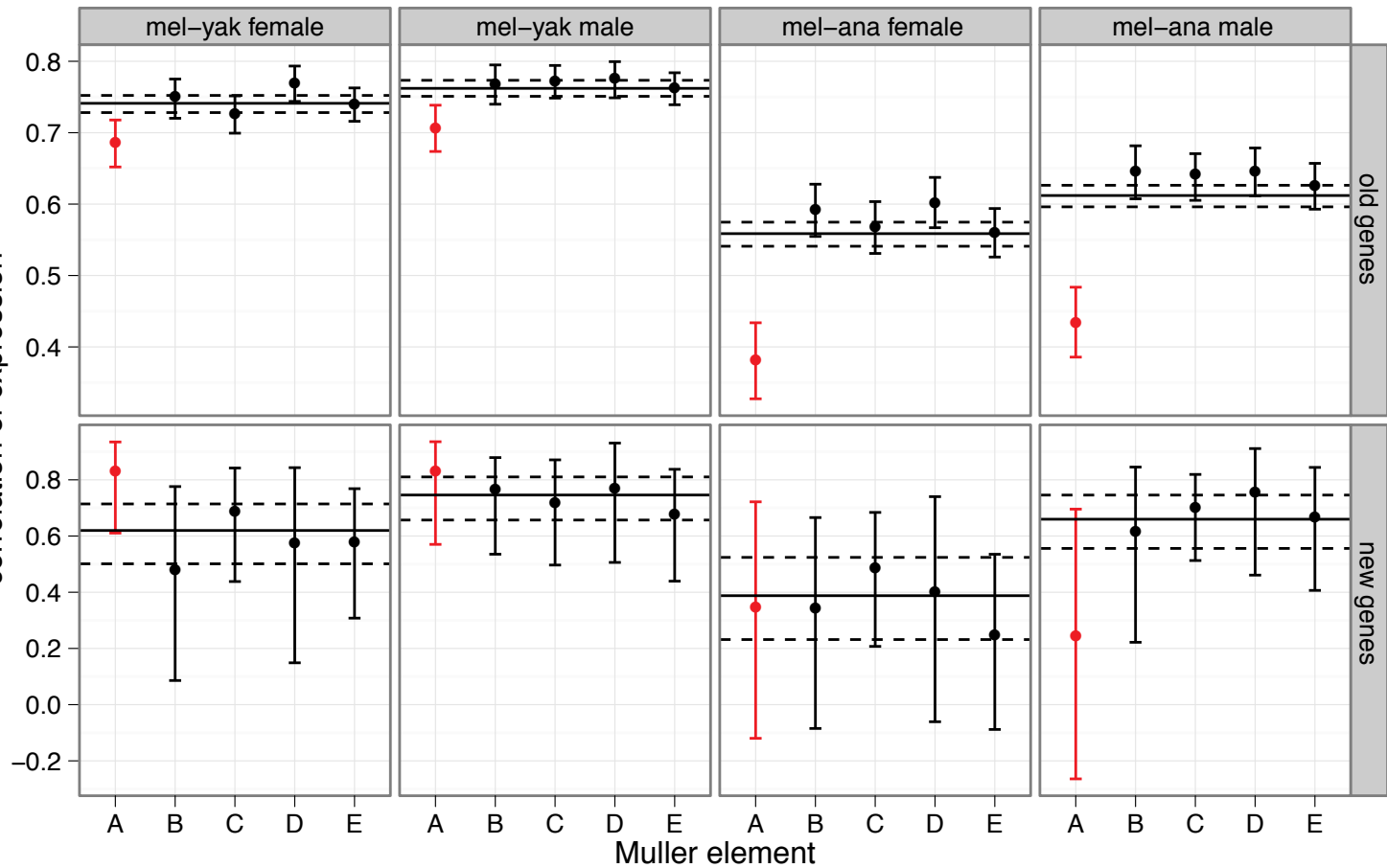

Supplement: Figure S8 — New genes do not exhibit faster-X evolution of gene expression Pairwise correlations of gene expression between D. melanogaster and D. yakuba (mel-yak) and D. melanogaster and D. ananassae (mel-ana) for genes on each chromosome arm. Correlations were calculated using genes shared by all species in the Drosophila genus and genes that arose along the lineage leading to D. melanogaster after the split with the Drosophila subgenus [61]. (PDF) [file pgen.1003013.s008.pdf]

### A. Female expression level

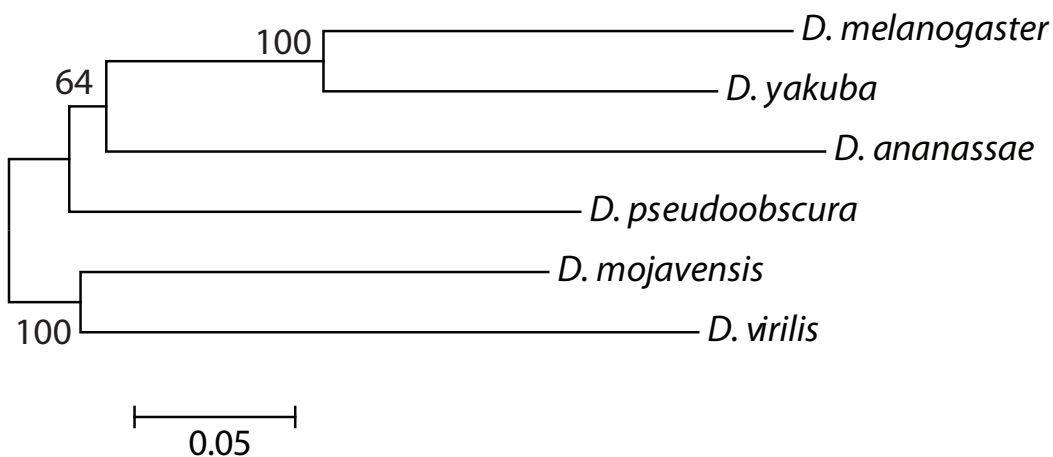

### B. Male expression level

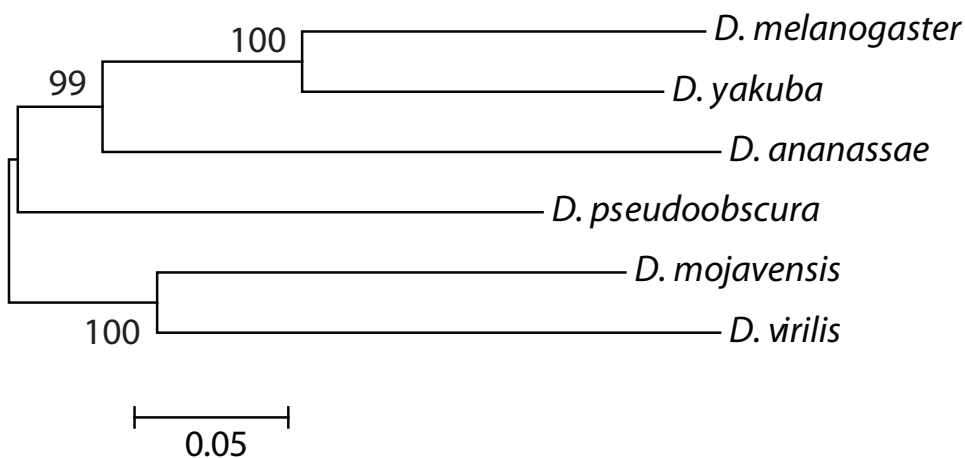

Supplement: Figure S9 — Phylogenetic reconstruction using the correlation of expression. We reconstructed the evolutionary relationships of the six species using the pairwise correlation of expression levels in (A) females and (B) males. A distance matrix of was used to build the phylogenies using the Fitch and Margoliash [115] method. We bootstrap sampled the genes 1000 times to estimate the support for each node, and the percent of bootstrap replicates supporting each node is given on the tree. (PDF) [file pgen.1003013.s009.pdf]

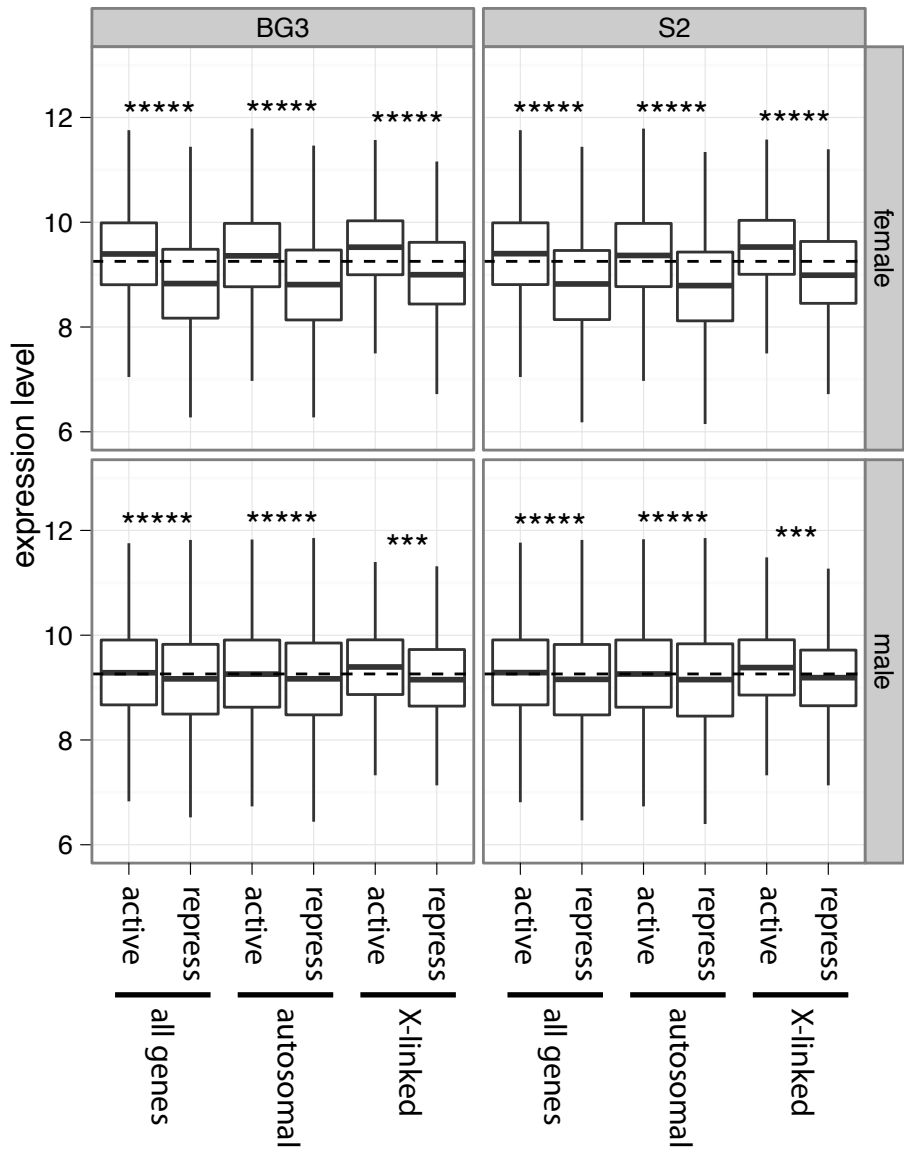

Supplement: Figure S10 — Expression levels of genes in transcriptionally active and repressive chromatin Boxplots show the distribution of expression levels for D. melanogaster genes in transcriptionally active and repressive (repress) chromatin. Horizontal dashed lines represent the genome-wide expression level. Chromatin states were measured in two cell lines (BG3 and S2), and expression levels were measured in either females or males. In addition, genes were divided into those that are autosomal and those that are X-linked. Significant differences between the expression levels of genes in active and repressive chromatin are indicated by asterisks (*** , ***** ). (PDF) [file pgen.1003013.s010.pdf]

BG3

S2

expression divergence

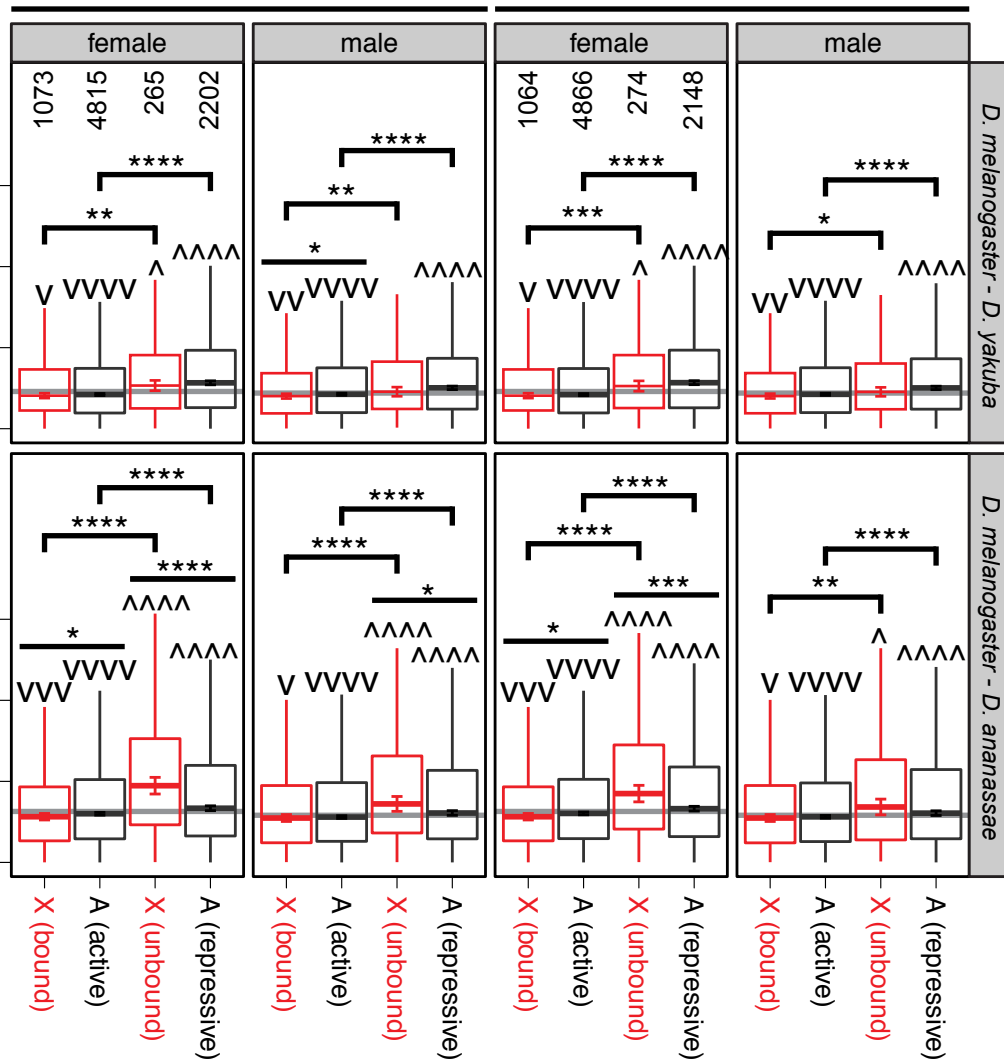

Supplement: Figure S11 — Faster expression evolution of un-dosage-compensated X-linked genes not associated with active chromatin. This graph is the same as the one in Figure 6 except that X-linked genes are now divided into those that are both bound by the DCC and in active chromatin or unbound by the DCC and in repressive chromatin. (PDF) [file pgen.1003013.s011.pdf]

correlation ( $\rho$ ) between  
expression divergence and  $\tau$

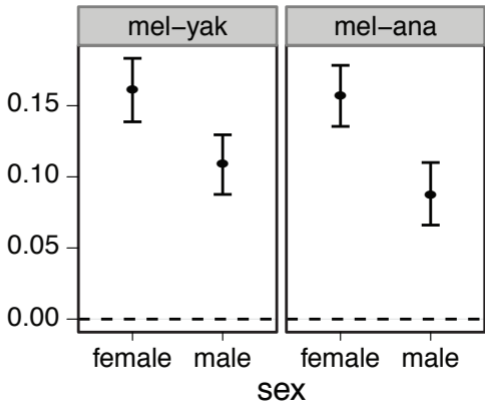

Supplement: Figure S12 — Narrowly expressed genes have greater expression divergence. Spearman's rank order correlation between and expression divergence is plotted, along with the 95% CI of the correlation. Larger values of indicate narrower expression breadth. Divergence was measured between D. melanogaster (mel) and both D. yakuba (yak) and D. ananassae (ana) using measurements from both females and males. The dashed line indicates the null expectation of no correlation. (PDF) [file pgen.1003013.s012.pdf]

**A**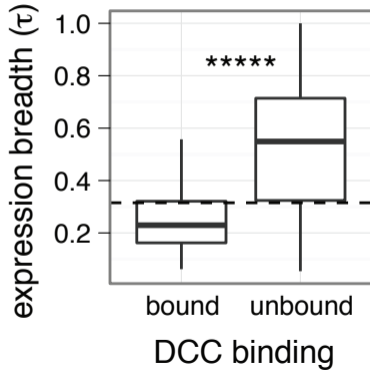**B**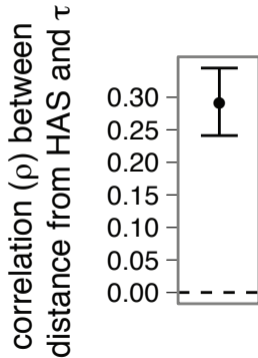

Supplement: Figure S13 — DCC bound genes tend to be broadly expressed. X-linked genes were divided into those that are bound and unbound by the DCC. (A) Expression breadth () was compared between bound and unbound genes using a Mann-Whitney U test (***** ). Larger values of indicate narrower expression breadth. (B) SpearmanÕs rank order correlation between distance from the nearest HAS and is plotted, along with the 95% CI of the correlation. The dashed line indicates the null expectation of no correlation. (PDF) [file pgen.1003013.s013.pdf]

**A**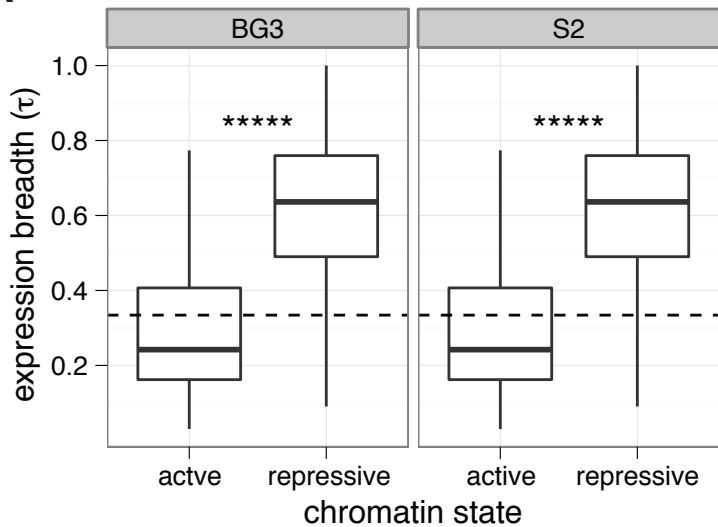**B**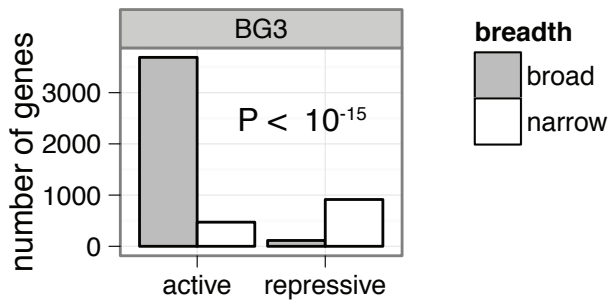

Supplement: Figure S14 — Genes in repressive chromatin are more narrowly expressed. Genes were divided into those that are in transcriptionally active and repressive chromatin using data from two cell lines: BG3 and S2. (A) Expression breadth () was compared between genes in active and repressive chromatin using a Mann-Whitney U test (***** ). Larger values of indicate narrower expression breadth. (B) Genes were additionally divided into those that are broadly and narrowly expressed, and the counts of genes in each expression breadth and chromatin state are plotted for data collected in BG3 cells (S2 data are available in Figure 7). Fisher's exact test was used to determine if there is a significantly non-random distribution of genes in the four classes. (PDF) [file pgen.1003013.s014.pdf]

# BG3 cells

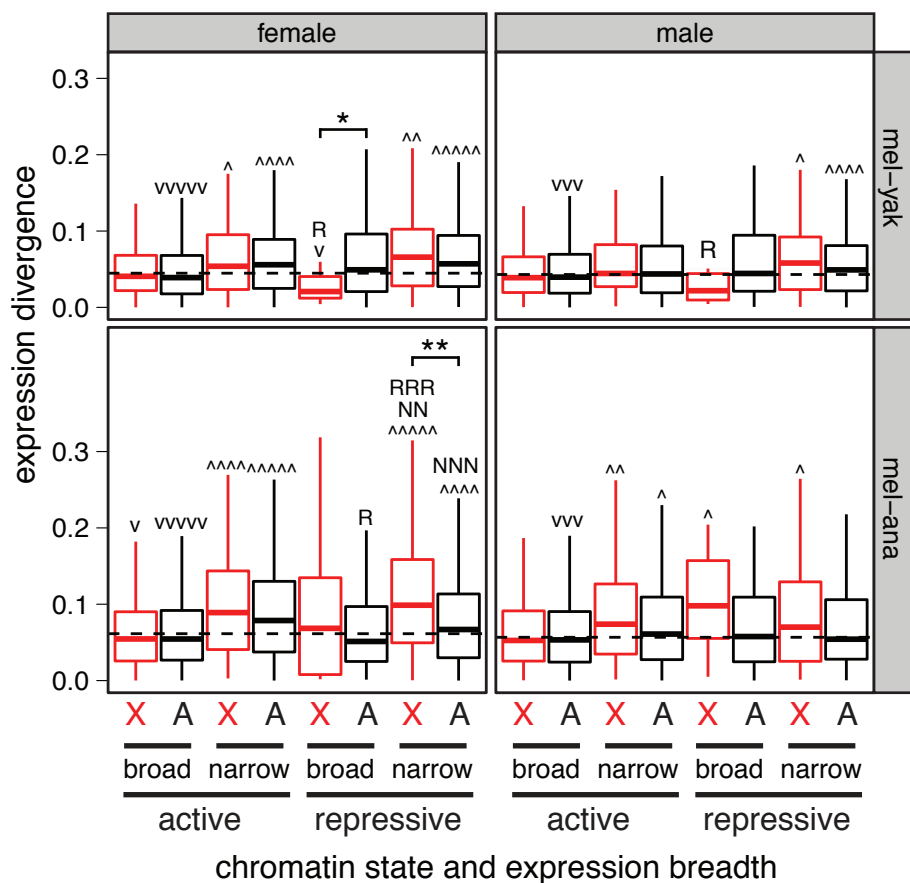

# S2 cells

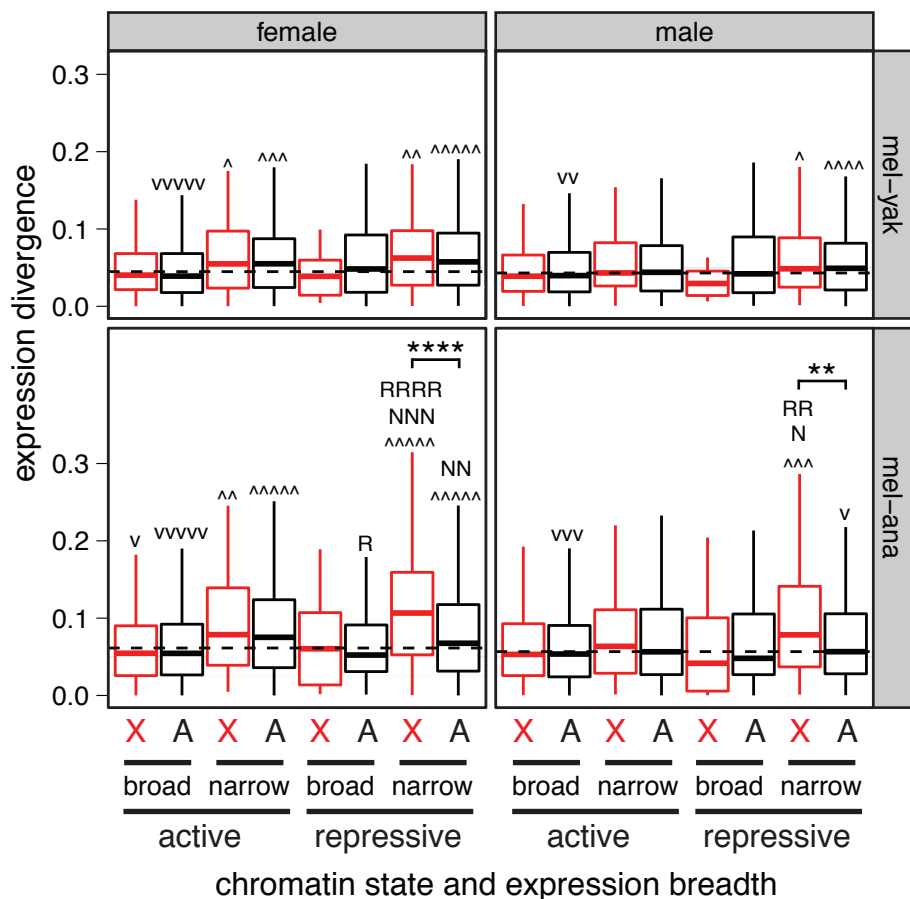

Supplement: Figure S16 — The faster-X effect is limited to narrowly expressed genes in transcriptionally repressive chromatin, with chromatin environment measured in BG3 cells. This figure is the same as Figure 8 and Figure S15 except that genes with the lowest 5% expression levels were excluded. (PDF) [file pgen.1003013.s016.pdf]

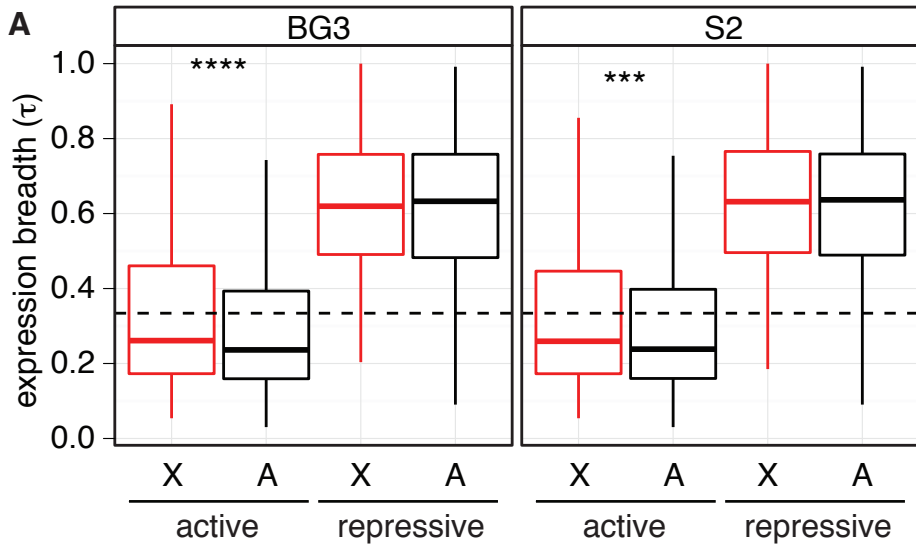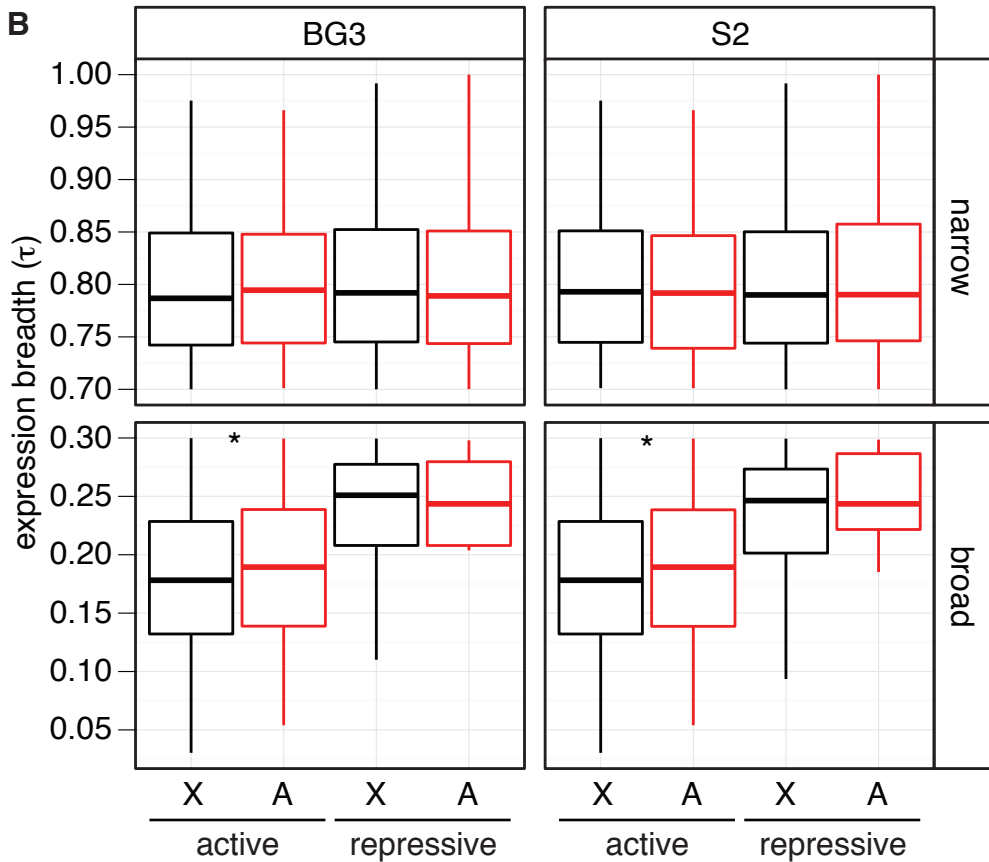

Supplement: Figure S17 — Expression breadth, chromatin environment, and X-linkage. The expression breadth () of genes in transcriptionally active and repressive chromatin on the X chromosome and autosomes is plotted. Larger values of indicate narrower expression breadth. Chromatin state was inferred in BG3 and S2 cells. Significant differences between X-linked and autosomal genes in the same chromatin context are indicated by asterisks (* , *** , **** ). (A) Median expression breadth across the entire genome is indicated by a dashed line. (B) Genes were additionally divided into narrowly and broadly expressed. (PDF) [file pgen.1003013.s017.pdf]

## BG3 cells

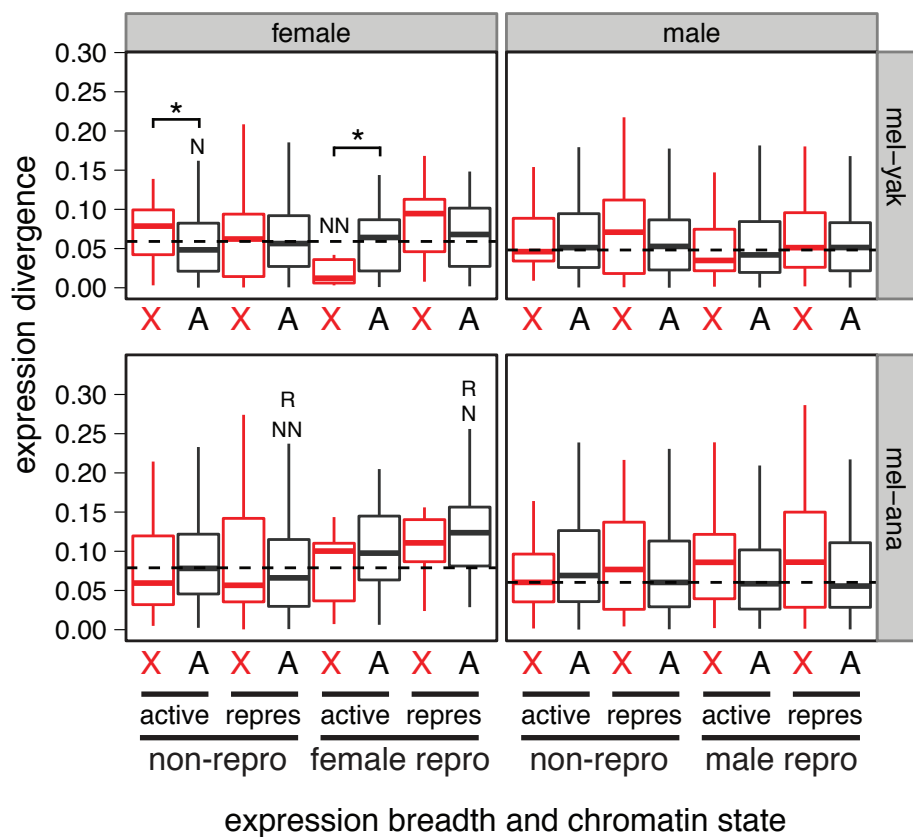

## S2 cells

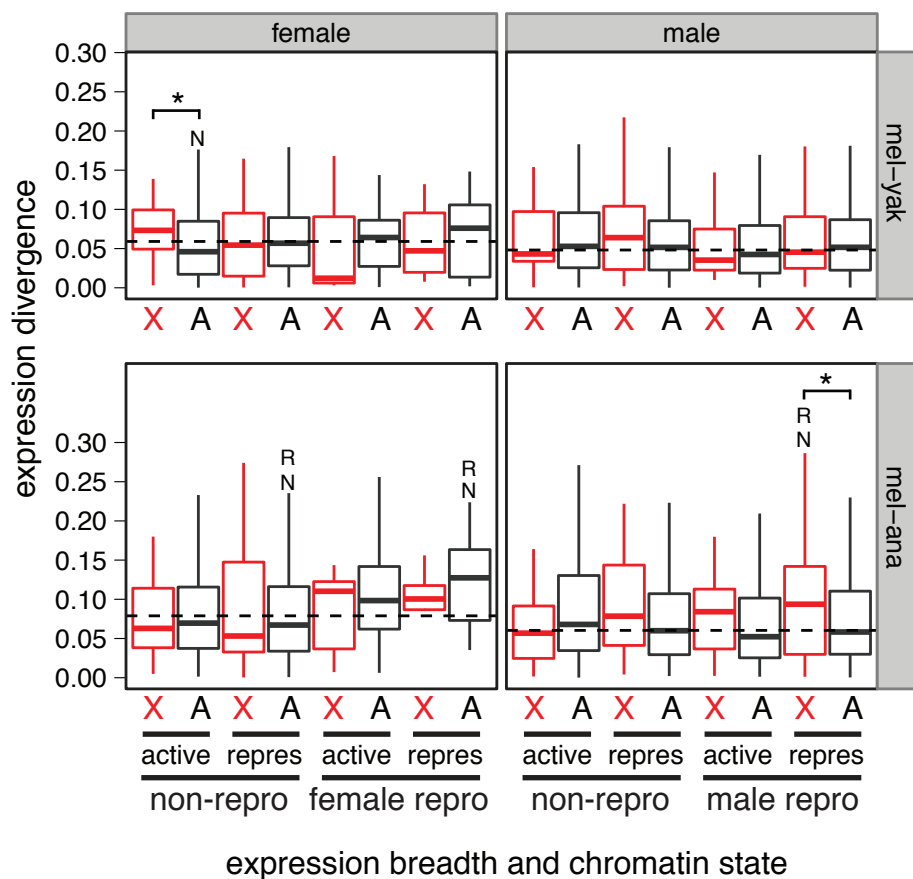

Supplement: Figure S18 — No faster-X effect for genes narrowly expressed in female reproductive tissues. Boxplots show the pairwise divergence in expression between 1∶1∶1 orthologs in the D. melanogaster (mel) and D. yakuba (yak) or D. ananassae (ana) genomes measured in whole females and males (see Figure 6). Only genes that are narrowly expressed in non-reproductive tissues (non-repro), female reproductive tissues (female repro), or male reproductive tissues (male repro) are included. X-linked (X, red) and autosomal (A) genes were assigned to transcriptionally active and repressive chromatin based on the results of experiments in BG3 (top) and S2 (bottom) cells. The horizontal dashed line indicates the genome-wide average pairwise divergence for narrowly expressed genes. Subsets of narrowly expressed genes whose pairwise divergence is significantly different than all other narrowly expressed genes are marked (N). The same was done for subsets of narrowly expressed genes that reside in repressive chromatin (R). Significant differences between X-linked and autosomal genes in the same chromatin state and with the same expression breadth are marked with asterisks. Mann-Whitney U tests were used to assess significant differences (one symbol = , two symbols = , three symbols = , four symbols = , five symbols = ). (PDF) [file pgen.1003013.s018.pdf]
