# Supplementary material for: Faster-X Evolution of Gene Expression in Drosophila
Source: PLoS Genet. 2012 Oct 11;8(10):e1003013. doi: 10.1371/journal.pgen.1003013 (PMC3469423; doi:10.1371/journal.pgen.1003013)
Supplement: Figure S15 — The faster-X effect is limited to narrowly expressed genes in transcriptionally repressive chromatin, with chromatin environment measured in BG3 cells. This figure is the same as Figure 8 except chromatin state was measured in BG3 cells. (PDF) [file pgen.1003013.s015.pdf]

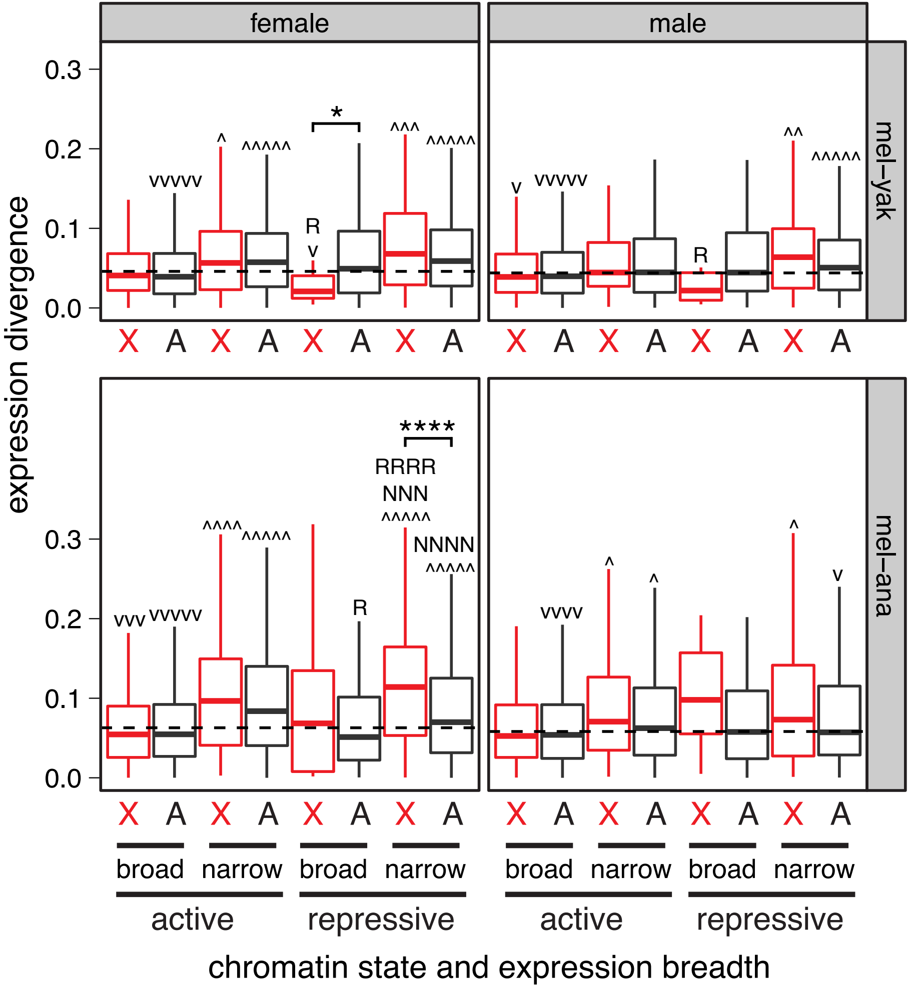
female
male
mel–yak
mel–ana
expression divergence
0.0
0.1
0.2
0.3
*
****
vvvvv
^
^^^^^
R
v
^^^
v
^^
RRRR
NNN
^^^^^
NNNN
^^^^
vvv
vvvv
X
A
broad
narrow
active
repressive
chromatin state and expression breadth
