# Supplementary material for: Faster-X Evolution of Gene Expression in Drosophila
Source: PLoS Genet. 2012 Oct 11;8(10):e1003013. doi: 10.1371/journal.pgen.1003013 (PMC3469423; doi:10.1371/journal.pgen.1003013)
Supplement: Table S1 — DCC binding and chromatin state. Genes were called as bound by the DCC if they were bound in S2 cells (SL2); either SL2, larval wing imaginal disc, or late embryonic cells (Any); or based on a separate analysis of the data (Bachtrog) [48]. Chromatin states (chr.state) were inferred in S2 cells or BG3 cells. The number of genes in each DCC binding and chromatin state class (num_genes) were tested for independence using Fisher's exact test, and the P value of this test is reported (FET.p). (PDF) [file pgen.1003013.s019.pdf]

DCC.cell.line	DCC	chr.cell.line	chr.state	num_genes	FET.p
SL2	bound	S2	active	442	5.39 x 10^-50
SL2	unbound	S2	active	622	
SL2	bound	S2	repressive	3	
SL2	unbound	S2	repressive	271	
Any	bound	S2	active	694	8.11 x 10^-91
Any	unbound	S2	active	370	
Any	bound	S2	repressive	7	
Any	unbound	S2	repressive	267	
Bachtrog	bound	S2	active	769	1.05 x 10^-97
Bachtrog	unbound	S2	active	295	
Bachtrog	bound	S2	repressive	15	
Bachtrog	unbound	S2	repressive	259	
SL2	bound	BG3	active	443	1.24 x 10-49
SL2	unbound	BG3	active	630	
SL2	bound	BG3	repressive	2	
SL2	unbound	BG3	repressive	263	
Any	bound	BG3	active	696	1.19 x 10^-90
Any	unbound	BG3	active	377	
Any	bound	BG3	repressive	5	
Any	unbound	BG3	repressive	260	
Bachtrog	bound	BG3	active	767	8.49 x 10^-90
Bachtrog	unbound	BG3	active	306	
Bachtrog	bound	BG3	repressive	17	
Bachtrog	unbound	BG3	repressive	248	
